# Chimeric forecasting: combining probabilistic predictions from computational models and human judgment

**DOI:** 10.1186/s12879-022-07794-5

**Published:** 2022-11-10

**Authors:** Thomas McAndrew, Allison Codi, Juan Cambeiro, Tamay Besiroglu, David Braun, Eva Chen, Luis Enrique Urtubey De Cèsaris, Damon Luk

**Affiliations:** 1 College of Health, Lehigh University, Bethlehem, PA USA; 2 Metaculus, Santa Cruz, CA USA; 3 Department of Epidemiology, Mailman School of Public Health, Columbia University, New York, USA; 4 Massachusetts Institute of Technology, Cambridge, MA USA; 5 Department of Psychology, Lehigh University, Bethlehem, PA USA; 6 Good Judgment Inc., New York, NY USA

## Abstract

Forecasts of the trajectory of an infectious agent can help guide public health decision making. A traditional approach to forecasting fits a computational model to structured data and generates a predictive distribution. However, human judgment has access to the same data as computational models plus experience, intuition, and subjective data. We propose a chimeric ensemble—a combination of computational and human judgment forecasts—as a novel approach to predicting the trajectory of an infectious agent. Each month from January, 2021 to June, 2021 we asked two generalist crowds, using the same criteria as the COVID-19 Forecast Hub, to submit a predictive distribution over incident cases and deaths at the US national level either two or three weeks into the future and combined these human judgment forecasts with forecasts from computational models submitted to the COVID-19 Forecasthub into a chimeric ensemble. We find a chimeric ensemble compared to an ensemble including only computational models improves predictions of incident cases and shows similar performance for predictions of incident deaths. A chimeric ensemble is a flexible, supportive public health tool and shows promising results for predictions of the spread of an infectious agent.

**Supplementary Information:**

The online version contains supplementary material available at 10.1186/s12879-022-07794-5.

## Introduction

Forecasts of the transmission and burden of COVID-19 provide public health officials advance warning that allows them to make informed decisions about how to modify their response to the pandemic [1–9]. The COVID-19 pandemic has caused economic burdens to the US, overwhelmed hospitals with ill patients, and further highlighted social inequity and inequalities in access to healthcare [10–15].

In response, several organized modeling efforts were started to give public health officials as up to date information as possible about the trajectory of COVID-19 in the US and in Europe [7, 16–18].

The US COVID-19 Forecast Hub is a unified effort to house probabilistic forecasts of incident cases, deaths, and hospitalizations due to COVID-19 in a single, centralized repository [16, 19]. The goal of this repository is to collect, combine, and evaluate forecasts of the trajectory of COVID-19 and communicate these forecasts to the public and to public health officials at the state and federal level [20]. This repository is not meant to include all possible forecasting targets related to COVID-19, and models not included in the COVID-19 Forecast Hub have forecasted vaccine safety, efficacy, and timing, conditional trajectories of COVID-19 given public health action, time-varying $R_{0}$ values, hospital bed requirements, among others [21–27]. The strength of the COVID-19 Forecast Hub is it’s ability to store, evaluate and communicate forecasting efforts systematically and focus modeling efforts that process objective, reportable data.

In addition to the US COVID-19 Forecast Hub, there are COVID-19 hubs that collect computational forecasts for Europe and specifically for Germany and Poland [16–18]. The majority of models submitted to these hubs are computational: statistical or dynamical models trained on structured data.

Statistical models build a forecast by leveraging correlations between the current trajectory of COVID-19 and a set of covariates [28–37]. Traditional data sources that were used to train models include historical counts of incident cases, deaths, and hospital admissions. A subset of models also train on novel sources of data such as self-reported COVID symptom rates and the rate of visits to a doctor, data related to mobility or contact among individuals, and social media data [38–41].

Dynamical models first pose a deterministic relationship for how an outbreak is expected to evolve and then typically assume that the observed data follows a random process to account for uncertainty between the (conjectured to be true) deterministic process and what is reported [42–44]. The most common dynamical models of the trajectory of COVID-19 extend compartmental models, models that assume individuals are in one of a finite set of states through the pandemic, to incorporate time varying reproduction numbers, multiple different data sources, and more complicated spatial structure [45–48]. Dynamical models often excel at long term forecasts and generating a predictive density over an epidemiological variable of interest in response to public health action or potential scenarios [47, 49–53].

Human judgment forecasting relies on the beliefs and activities of a crowd to generate (point or probabilistic) predictions over the possibilities of some future event. Below we present examples of three types of human judgment forecasting: prediction markets, incorporating passive human judgment data into a model, and collecting direct human judgment predictions.

Prediction markets have been developed to predict infectious diseases such as the 2009 swine flu, seasonal influenza, enterovirus, and dengue fever [54–56]. A prediction market provides participants an initial amount of “money” to spend on future events and allows participants to place higher bids on events they think are more likely to occur. After bidding is complete, a model maps the “market price” for each event to a probability which is interpreted as the crowd’s belief that event will occur [57]. Prediction markets rely on a large and diverse participant pool and the model that connects market price to predictive probability to make accurate predictions [58, 59].

Passive human activity and behavior from social media outlets like Twitter and Facebook, and internet search history have been used as inputs to a model and have shown improved accuracy compared to a model that uses only epidemiological data for infectious agents like influenza, dengue fever, ZIKA, and COVID-19 [60–65]. Most models (i) extract features from these social media outlets, (ii) transform the extracted social media data and include objective epidemiological data, and (iii) train a predictive model on this combination of objective, subjective data. Models using social media data are usually statistical or machine learning models, exploiting correlations between these data sources and the target of interest.

Direct predictions—either point predictions or probability densities—of the trajectory of an infectious agent have been elicited from individuals and aggregated for diseases such as influenza and COVID-19 [21, 66–68]. Point forecasts have been elicited from experts from platforms like Epicast [67]. Epicast asks participants to predict the entire trajectory of influenza-like illness (ILI), a marker for the severity of seasonal influenza, by viewing the current ILI time series and then drawing a proposed trajectory from the present week to the end of the influenza season. The aggregate model assigns a probability to an ILI value belonging in the bounded interval $[x,x+\delta ]$ as the proportion of individual trajectories that fall within those bounds. The Epicast model was routinely one of the top performing models among several computational models submitted to the CDC sponsored FluSight challenge [67].

Three projects to date have collected direct, probabilistic predictions from humans about the transmission and burden of the COVID-19 pandemic [66, 68, 69]. As early as February 2020, human judgment platforms have made predictions of the trajectory of COVID-19 by enrolling experts in the modeling of infectious disease and asking them questions related to reported and true transmission, hospitalizations, and deaths due to SARS-CoV-2 [66]. Experts were also asked to make predictions of transmission conditional on future public health actions. An equally weighted average of expert predictions was used to combine individual predictions into consensus predictions and reports from this work were generated from February 2020 to May 2020. This work found that, although there was considerable uncertainty assigned to confirmed cases and deaths, a consensus of expert predictions was robust to poor individual predictions, able to make accurate predictions of confirmed cases one week into the future, and gave an early warning signal of the severity of SARS-CoV-2. The second project compared predictions of rates of infection and number of deaths between those who were considered experts and laypeople in the United Kingdom [69]. Participants were asked to assign a 12.5th and 87.5th percentile to four questions related to COVID-19—one question with ground truth and three with estimated values for the truth. Expert predictions were more accurate and calibrated than non-expert predictions, however expert predictions still underestimated the impact of COVID-19. A third project solicited from experts in statistics, forecasting, and epidemiology direct predictions of one through four week ahead incident and cumulative cases and deaths for Germany and Poland (at the national level) and aggregated these predictions into a “crowd forecast” [68]. The crowd was able to produce more accurate, calibrated—as measured by the weighted interval score—predictive forecasts of cases in both countries compared to computational models, however computational models made more accurate predictions of deaths.

Human judgment predictions have been applied to a numerous number of fields beyond infectious disease and interested readers can find comprehensive reviews on the status and applications of human judgement forecasting [21, 70, 71]. Select foundational works on aggregating human judgment may be found in the following citations [71–75].

We propose an ensemble algorithm designed to generate forecasts of the trajectory of an infectious agent by combining direct, probabilistic predictions from computational models and human judgement models. We call this ensemble a chimeric ensemble. There exists in the literature many recipes for combining computational models and models of human judgment, and we include here only a small number of past works on this topic that we feel will provide the reader an introduction to the discipline [76–85].

In this first hypothesis-generating work we: (i) explore the advantages and challenges when combining computational and human judgment models, (ii) compare the performance of a chimeric ensemble to a computational model only ensemble on six forecasts of incident cases and six forecasts of incident deaths due to COVID-19 at the US national level between January 2021 and June 2021, (iii) compare and contrast an algorithm that assigns different weights to computational models and human judgement based on past performance to an equally weighted combination of models, and (iv) finally shows how a chimeric ensemble can leverage human judgement data to improve predictive performance of an outbreak.

## Methods

### Forecasting logistics

#### Survey timeline

Six monthly surveys were sent to experts and trained forecasters from January to June 2021 on the Metaculus forecasting platform https://www.metaculus.com/ and five monthly surveys from February to June 2021 were sent to the Good Judgment Open (GJO) platform https://www.gjopen.com/. Participants had approximately ten days to add probabilistic predictions, and were encouraged to include a rationale alongside their quantitative forecasts to provide insight into how they made their predictions. Participants on both platforms were allowed to revise their original predictions as many times as they wished between when the survey was open and when it closed (often ten days later). During the course of all six surveys, participants could revisit their past predictions but could no longer revise predictions for those surveys that were closed. A list of survey open and close times, questions that were asked, and how the truth was determined for each question can be found in supplement A.

We note that the survey period from January to June, 2021 was during a time when incident cases and deaths was declining which may limit how our analysis generalizes to epidemic trajectories that increase or increase, peak, and then decrease.

#### Forecaster elicitation

All subscribers to the Metaculus platform and to the GJO platform were invited to make anonymous predictions of epidemiological targets related to COVID-19. Subscribers to Metaculus were sent email invitations and all questions related to this project were grouped together and posted on the Metaculus website as a tournament titled *Consensus Forecasting to Improve Public Health: Mapping the Evolution of COVID-19 in the U.S.* which can be found at https://pandemic.metaculus.com/questions/?search=contest:consensus--forecasting. Subscribers to GJO were invited to participate via email and questions for this project were posted on the GJO website as “Featured Questions”. A convenience sample of 16 experts were invited to participate on the Metaculus platform. We defined an expert as one who has several years of experience in the study or modeling of infectious disease and have kept up to date on scientific literature, and public health efforts related to COVID-19.

Both the Metaculus and GJO platforms offer training and prediction resources on their websites (https://www.metaculus.com/help/prediction-resources/ and https://goodjudgment.com/services/online-training/) that allows a subscriber to familiarize themselves (i) with how to make calibrated and accurate predictions and (ii) how to use the tools and features of the platform.

Forecasters on Metaculus and Good Judgment receive, for each question they answer on the website, immediate feedback from a visualization of the present consensus forecast and longer term feedback by receiving an email when the ground truth for a question resolves and a score that determines the accuracy of their prediction for a specific question.

#### How predictions were collected from humans

Forecasters submitted monthly predictions in a format that depended on if they used the Metaculus platform or the Good Judgment Open platform.

Participants on Metaculus generate predictions over a continuous bounded interval as a combination of up to five logistic distributions (Additional file 1: Fig. S1). When a participant decides to form a prediction they are presented with a single logistic distribution and a slider bar underneath this distribution. The slider contains a square indicating the distribution median and two circles to the left and right of the square that help identify the distribution’s 25th and 75th quantiles. Participants can shift this distribution left, over smaller values, or right, over larger values, by moving the square and they can scale this distribution by expanding or contracting the circles to the left and right of the square. If a participant decides to include a second (third, fourth, and fifth) logistic distribution they can select “add component”. A second predictive density is overlaid over the first and the participant can control that second density by using a second slider that appears below the first. In addition to the two sliders, an additional two slider bars appear that allow the participant to assign weights to the first and second (third, fourth, fifth) predictive densities.

Participants on GJO assign probabilities to a set of intervals $I_{1},I_{2},\cdots ,I_{n}$ that partition an open interval Additional file 1: Fig. S2). For each interval $I_{i}$, participants are presented a slider bar controlling the probability assigned to $I_{i}$ and that can be at minimum zero and maximum one. To the right of each slider bar is a text box that contains the current probability the participant has assigned to $I_{i}$. The probabilities assigned to all intervals must sum to one, and as a participant selects probabilities to assign to each interval the total probability is computed and displayed. A participant can only submit a probability distribution when the total probability equals one.

### COVID-19 Forecast Hub

The COVID-19 Forecast Hub collects prospective forecasts of the trajectory of COVID-19 in the United States from more than 80 computational models [16, 20, 86]. Forecasts of weekly incident cases are produced at the national, state, and county level, and forecasts of weekly incident and cumulative deaths and daily hospitalizations are produced at the national and state levels. Forecasts of cases are submitted to the COVID-19 Forecast Hub as a set of 7 quantiles and forecasts of deaths are submitted as a set of 23 quantiles. Models produce predictions of weekly cases and deaths one, two, three, and four weeks ahead. A GitHub repository (https://github.com/reichlab/covid19-forecast-hub) is used to keep track of individual submissions and an ensemble model.

### Human judgement forecasting targets

Members of the Metaculus and GJO crowd were asked to predict the number of incident cases and incident deaths due to COVID-19 that would be observed at the US national level over the course of one epidemic week. These “core” questions were asked for all six surveys, were presented to humans in the same format for all six surveys, and were meant to match, as much as possible, to the corresponding forecast targets used by the COVID-19 Forecast Hub.

In addition to these core questions, we asked the Metaculus crowd only extra questions of public health relevance. Example questions include the cumulative number of first and full dose vaccinations by a given date, cumulative deaths by year end, the 7-day moving average of the percent of B.1.1.7 in the US, and the incident number of weekly hospitalizations. A list of all questions asked throughout the six surveys can be found in the supplement (Additional file 1).

### Matching COVID-19 Forecast Hub and human judgement forecasting targets

How questions were posed to human judgement crowds and how the truth was determined for questions related to incident cases and incident deaths at the US national level matched how the ground truth was determined by the COVID-19 Forecast Hub. When we described the resolution criteria for forecasts of incident cases and deaths, we matched, as close as possible, the ground truth document sent to modeling teams who submit computational forecasts to the COVID-19 Forecast Hub (technical readme for COVID-19 Forecast Hub: https://github.com/reichlab/covid19-forecast-hub/blob/master/data-processed/README.md).

**Fig. 1:**
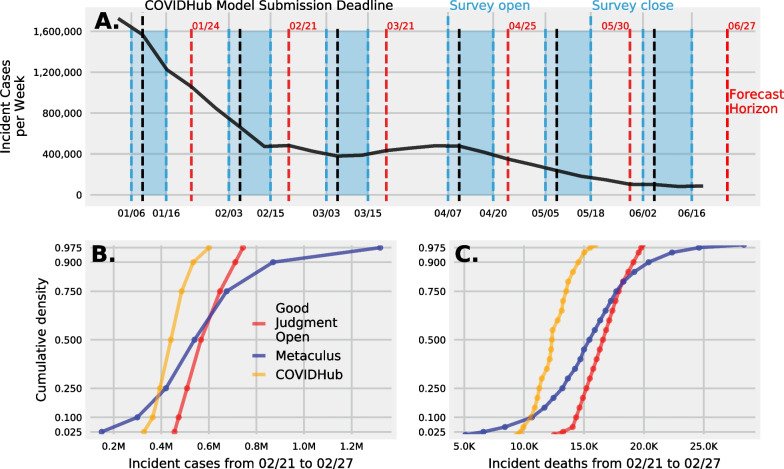
**A** A timeline of the six surveys that collected human judgment predictions from January to June of 2021, showing when surveys were open and closed (blue dashed lines), when computational predictions submitted to the COVID-19 Forecast Hub were due (black dashed line), human judgment predictions excluded in formal analysis (dark blue), for what week each forecast was made (red dash line), and the reported number of weekly incident COVID-19 cases at the US national level (black solid line). ** B** Forecasts of weekly incident cases submitted to the COVID-19 Forecast Hub (orange) were formatted as seven quantiles, and we similarly formatted human judgment predictions from Metaculus (blue) and Good Judgment Open (red). **C** Forecasts of weekly incident deaths submitted to the COVID-19 Forecast Hub were formatted as twenty three quantiles and we formatted human judgment predictions the same. We collected more than 3000 original and revised human judgement predictions of incident cases and deaths of the spread of SARS-CoV-2 and burden of COVID-19 in the US

The COVID-19 Forecast Hub allows computational forecasts to be submitted at any time, but only computational forecasts that are submitted on Mondays of each week are included in the weekly COVID-19 forecast hub ensemble. Each survey sent to Metaculus and GJO crowds was open for submission before a COVID-19 Forecast Hub due date. In January surveys closed six days after the Monday due date, in February and March surveys closed on a Monday deadline, in April and May surveys closed one day after a COVID-19 Forecast Hub due date, and in June two days after a due date. Individual predictions submitted to Metaculus and to GJO were cut at the same due date as the one asked of computational models submitted to the COVID-19 Forecast Hub  Fig. 1A. Counts of the number of computational and human judgement models can be found in supplemental III. The goal with cutting individual predictions at the same time as computational model was for a fair comparison, and a fair combination of computational and human judgement forecasts.

### Forecast scoring

Individual, consensus, ensemble, and chimeric forecasts were scored using the weighted interval score (WIS) over *K* central quantiles [87].

$$\begin{aligned} \text {WIS}_{\alpha _{\{0:K\}}}(F,y) = \frac{1}{K + 1/2} \left( w_0 \times |y-m| + \sum _{k=1}^{K} \{w_k \times \text {IS}_{\alpha _k}(F,y)\} \right) \end{aligned}$$

where the interval score $(\text {IS}_{\alpha _{k}})$ is

$$\begin{aligned} \text {IS}(\alpha )(F,y) = (u-l) + \frac{2}{\alpha } (l-y) 1\!\!1 (y<l) + \frac{2}{\alpha } (y-u) 1\!\!1 (y>u) \end{aligned}$$

and where *F* is a predictive cumulative distribution function, $1\!\!1(x)$ is an indicator function, the value *u* represents the $(1-\alpha /2)$ quantile of *F*, *l* represents the $\alpha /2$ quantile of *F*, and *m* represents the median or 0.50 quantile, and *y* is eventually reported truth [88]. Weight $w_{0}$ equals $\frac{1}{2}$ and $w_{k} = \frac{\alpha _{k}}{2}$.

The weighted interval score (and interval score) are negatively sensed—larger values indicate worse predictive performance compared to smaller values. The best possible weighted interval score is zero and the worst possible weighted interval score is positive infinity.

WIS is a discrete approximation of the continuous rank probability score

$$\begin{aligned} \text {CRPS}(F,y)= \int _{-\infty }^{\infty } \{F(x) - 1(x \ge y)\}^2 \,dx \end{aligned}$$

where the WIS score converges to the same value as the $\text {CRPS}$ as the number of equally spaced intervals (*K*) increases given a fixed cumulative density *F* and true value *y* [87].

The WIS is the score adopted by the Centers for Disease Control and Prevention (CDC) to evaluate forecasts of incident cases, deaths, and hospitalizations submitted as a set of set of central quantiles.

The WIS and CRPS are examples of negatively sensed proper scoring rules [88, 89]. A negatively sensed proper scoring rule is a function *S* that takes as input a density *F* and true value *y* and returns a non-negative real number that is minimized when the input density *F* is distributed the same as the true data generating process *Y* that produced the true, realized value *y* [88, 89].

### Consensus algorithm strategies

#### Data setup

Ensemble forecasting of infectious targets involves three related data sets: (i) data collected about epidemiological quantities of interest, ${\mathcal {D}}$, (ii) predictive densities over these targets submitted by individual models (either computational or human), *F*, and (iii) a score given to each model forecast about a collected data point, ${\mathcal {S}}$.

We suppose an epidemiological target, or quantity of interest (incident cases, deaths, etc.) at time *t* can be represented by a random variable $T_{t}$, and further assume true values ${\mathcal {D}} = [t_{1},t_{2},\cdots ,t_{N}]$ were generated by random variables $T_{1}, T_{2}, \cdots , T_{N}$ where $T_{t}$ is specific to a single target, point in time, and location. We make no additional assumptions about whether targets are dependent or independent and do not assume a specific distribution over potential target values.

A model produces a forecast for a target $T_{t}$ in the form of a set of *K* quantiles. We can organize forecasts *F* over all targets from *M* models that submitted *K* quantiles into a matrix where a forecast from a single model corresponds to one row and one column corresponds to a quantile about one target. For example, a forecast matrix with 3 models, *K* quantiles, and *T* targets can be formed as follows

$$\begin{aligned} F = \left[ \begin{array}{cccccccccccccc} \underline{\hbox {Model}}&{} &{}{\underline{\text {Target 1}}}&{}&{} &{}&{}{\underline{\hbox {Target 2}}}&{}&{}&{} \cdots &{} &{}{\underline{\hbox {Target T}}} &{}&{}\\ M_{1} | &{} q^{1}_{1,1} &{} q^{1}_{1,2} &{} \cdots &{} q^{1}_{1,K} &{} q^{1}_{2,1} &{} q^{1}_{2,2} &{} \cdots &{} q^{1}_{2,K} &{} \cdots &{} q^{1}_{T,1} &{} q^{1}_{T,2} &{} \cdots &{} q^{1}_{T,K} \\ M_{2} | &{} q^{2}_{1,1} &{} q^{2}_{1,2} &{} \cdots &{} q^{2}_{1,K} &{} q^{2}_{2,1} &{} q^{2}_{2,2} &{} \cdots &{} q^{2}_{2,K} &{} \cdots &{} q^{2}_{T,1} &{} q^{2}_{T,2} &{} \cdots &{} q^{2}_{T,K} \\ M_{3} | &{} q^{3}_{1,1} &{} q^{3}_{1,2} &{} \cdots &{} q^{3}_{1,K} &{} q^{3}_{2,1} &{} q^{3}_{2,2} &{} \cdots &{} q^{3}_{2,K} &{} \cdots &{} q^{3}_{T,1} &{} q^{3}_{T,2} &{} \cdots &{} q^{3}_{T,K} \\ \end{array}\right] \end{aligned}$$

No assumptions about a predictive density are placed on models beyond requiring a list of *K* quantile values.

A matrix ${\mathcal {S}}$ can also be built

$$\begin{aligned} {\mathcal {S}} = \begin{bmatrix} s_{1,1} &{} s_{1,2} &{} \cdots &{} s_{1,N}\\ s_{2,1} &{} s_{2,2} &{} \cdots &{} s_{2,N} \\ \vdots &{} &{} \ddots &{} \vdots \\ s_{M,1} &{} s_{M,2} &{} \cdots &{} s_{M,N} \\ \end{bmatrix} \end{aligned}$$

where the ${\mathcal {S}}_{ij}$ entry of this matrix, $s_{ij}$, corresponds to the score for model *i* about target *j*

#### Model combination and optimization

We chose to combine individual forecasts for our consensus and chimeric ensembles using a quantile average. We define a quantile average as a convex combination of individual forecast quantiles

$$\begin{aligned} f = F' \pi \end{aligned}$$

where *f* is a row vector of length *KN* and $\pi = [\pi _{1},\pi _{2},\cdots ,\pi _{M}]$ is a vector of length *M*. The weight vector $\pi$ is further constrained to have non-negative entries and to sum to one.

We will estimate weights for each model by finding a vector $\pi$ such that the ensemble forecast *f* minimizes in-sample mean WIS scores (*W*) over all targets with ground truth available. Given a sample of *T* realized true values ${\mathcal {D}} = [t_{1},t_{2},\cdots ,t_{T}]$

1 $$\begin{aligned} &\min _{f} \overline{ W(f)} \; \; \text {s.t.}\\&\pi ' 1\!\!1 = 1 \\&0 \le \pi _{m} \le 1 \end{aligned}$$

where $1\!\!1$ is a vector of ones, *W*(*f*) is a vector of WIS scores for *f*, and $\overline{W(f)}$ is the average WIS score for an ensemble density *f* over all targets. Because we choose weights $\pi$ to assign to out of sample probabilistic predictions which minimize an objective function, this process can be framed as a specific case of stacked generalization [90].

The algorithm we chose to optimize the weights assigned to computational and human judgment models is a population based optimization strategy called differential evolution. Differential evolution (DE) is a stochastic direct search method that is often robust to high dimensional parameter spaces and multi-modal objectives [91].

Given a set of *M* computational and human judgment forecasts at survey time *T*, the goal of this DE algorithm is to find a $M \times 1$ vector used to weight individual models that minimizes the mean WIS over all past survey time points for which we have the truth. To begin, DE chooses at random 4 $M \times 1$ vectors and evaluates the mean WIS score for each of the four weight vectors. At the next iteration each of the potential vector solutions, in turn, is compared to a new candidate vector solution. The candidate vector solution to be compared is generated by “mutation” and “crossover” (details can be found in [91]). Mutation and cross over have associated parameter values, and we chose a value of 0.8 for mutation and 0.9 for cross over. If the candidate solution reports a smaller mean WIS score than the original vector, the original vector is replaced with this new solution. This iteration is complete after all original solutions have been compared to new candidate solutions. Then the next iteration starts. All solutions were normalized by dividing the $M \times 1$ potential vector solution by the sum of all the entries to guarantee the final, minimal solution assigned weights that sum to one. Differential evolution was implemented by using the python package *mystic* [92, 93].

#### Methods to account for missing forecasts

We took three approaches to impute missing forecasts: (i) a complete case approach, (ii) an available forecast approach we call “spotty memory”, and (iii) an approach we call “defer to the crowd”.

The complete case approach combines models that have made forecasts for all targets asked for the present survey and all past surveys. If a model missed a forecast, past or present, they are removed from the ensemble. The “spotty memory” approach combines models if they have made forecasts for all targets in the present survey. If a model missed a forecast in the past they are still included. If a model missed a forecast for the present survey for either cases or deaths than they are removed from the ensemble. The “defer to the crowd” approach combines models that have made at least one forecast for any past or present survey. A model without a forecast for the present survey, but a model that has made a forecast on any previous survey is included and their present forecast is set to missing.

The complete case approach will have no missing forecasts, however we must impute missing forecasts for both the “spotty memory” and “defer to the crowd” approach. To impute a missing forecast, we considered each quantile a function of *K* quantiles submitted by *M* models about a single target. We only allow predictions of the same target to inform missing forecasts.

Define a matrix *Q* by selecting only those quantiles from *F* that correspond to a single target. The rows of *Q* correspond to models and the columns correspond to *K* quantiles where the smallest quantile is the first column, the second smallest quantile is the second column, up until quantile *K*. We denote $Q_{-k}$ as the matrix *Q* with column *k* removed and $Q_{k}$ as the $k^{\text {th}}$ column vector of *Q*.

Then we can impute $Q_{k}$ as a function *g* which takes as input $Q_{-k}$ and potentially some parameter set $\theta$

$$\begin{aligned} Q_{k} = g(Q_{-k},\theta ) \end{aligned}$$

We chose to test the following 5 approaches to impute missing forecasts: mean imputation, median imputation, bayesian ridge regression, decision tree regression and extremely randomized trees (see Table 1 for a summary of these methods).

**Table 1:** Five procedures were chosen to impute missing forecasts

Imputation technique	G	Summary
Mean	$I^{-1}\sum _{i}{q_{i,k}}$	Take the mean of all present quantiles where the set *I* is an index for present forecasts
Median	$\min _{x} \left\{ F(x)-1/2 \right\}$	Take the median of all present quantiles where *F* is the empirical cdf over all *I* quantiles
Bayesian Ridge regression	${\mathbb {E}}(X) \text { where } X\sim {\mathcal {N}}(Q_{-k}\beta ,\sigma ^{2})$ $\beta \sim {\mathcal {N}}(0,\uplambda ^{-1} I) \;\sigma ^{2} \sim \Gamma (\alpha ,\gamma )$	The matrix $Q_{-k}$ has two columns: a column of ones and a second column of quantiles from present forecasts.
Decision Tree regression	–	The missing quantile value is imputed by the mean of quantiles in the same partition.
Extremely Randomized Trees	–	Multiple decision trees $(D_{i})$ are fit to random subsets of quantiles and the missing forecast is imputed as the average over $D_{i}$.

Mean and median imputation only use information about a single quantile to impute missing forecasts, while the three regression approaches use all the quantiles from all present forecasts to impute missing forecasts

For the last three regression approaches, missing quantiles were imputed using a chained equation process. The chained equation process imputed missing values in four steps. Step one, replace missing quantiles in $Q_{k}$ with the mean over all present quantiles in column *k*. Step two, choose the column with the fewest missing values, set the values imputed with the mean back to missing. Step 3, impute missing values for column *k* using $g(Q_{-k},\theta )$. Step 4, repeat the above process on the quantile with the second fewest number of missing values. The above steps are iterated until convergence. We used the “IterativeImputer” function from scikit-learn to perform this chained equation imputation [94].

## Results

### Survey logistics and participation

A total of six surveys were run from January 2021 to June 2021. Each survey asked on average 7.5 questions related to national level incident cases, incident deaths, incident hospitalizations, the cumulative number of first dose and fully vaccinated individuals, and additional questions of immediate public health importance such as the proportion of sequences classified as B.1.17 among all sequenced viruses. A list of all questions asked for each survey can be found in Additional file 1: section A. At the end of each month a summary report was generated and posted online (summary reports can be found at the following link=https://github.com/computationalUncertaintyLab/aggStatModelsAndHumanJudgment_PUBL).

We collected from the Metaculus platform predictions from 68 unique members who made a total of 1062 original and revised predictions across all twelve questions related to cases and deaths. From GJO we collected predictions from 323 unique members who made 3319 original and revised predictions.

From the COVID-19 Forecast Hub we collected a total of 364 predictions of incident cases and incident deaths at the national level generated by 46 computational models between January and June of 2021. Computational models used a variety of techniques to build predictions of incident cases and deaths such as traditional statistical time series models like ARIMA and state space models, machine learning techniques such as deep artificial neural networks, and compartmental models. A list of the computational models included in this analysis can be found in supplement C.

The number of weeks between when a forecast was generated (the forecast date) and the week when the truth would be determined (the target end date) was 2 weeks for January, February, March, and April, and 3 weeks for May and June. There were more than one forecast date we could have chosen between the start and close date of each survey. We decided to chose the earliest forecast date that was the same as the COVID-19 forecast date (Fig. 1A.).

Analyses below focus on predictions of incident cases which were formatted as 7 quantiles: 0.025, 0.100, 0.250, 0.500, 0.750, 0.900, 0.975 (Fig. 1B.) and incident deaths which were formatted as 23 quantiles: 0.01, 0.025, quantiles from 0.05 to 0.95 in increments of 0.05, 0.975, and 0.99 at the national level (Fig. 1C.). These 12 predictions were made by both human judgment and computational models at overlapping times.

### Ensemble and individual performance

**Fig. 2:**
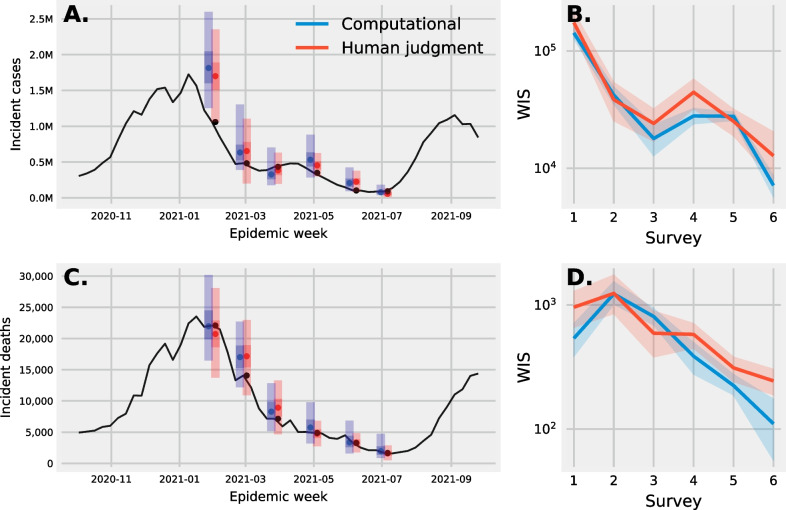
**A** Forecasts of weekly incident cases at the national level by an ensemble of computational models (blue) and ensemble of human judgement (red). The dot represents the median forecast and the shaded bars represent the 25th and 75th, and the 2.5th and 97.5th prediction intervals. ** B** A mean and 95% confidence interval of the weighted interval score (WIS) for forecasts of incident cases made by individual computational and human judgement models. **C** Forecasts of weekly incident deaths and forecasts from computational models and human judgement. **D** Mean and 95% confidence intervals of the WIS for individual predictions of incident deaths. Though individual human judgement forecasts tend to perform worse than computational models, a human judgement ensemble performed similar to an ensemble of computational models for predictions of both cases and deaths over a 6 month period

An ensemble of human judgment models made similar two and three week ahead predictions of weekly incident cases and deaths at the national level when compared to a computational ensemble (Fig. 2A, C) despite individual human judgement predictions performing slightly worse on average (Fig. 2B, D).

The median prediction of incident cases was closer to the truth on more occasions for human judgement compared to computational models (Fig. 2A). Human judgement and computational ensembles both overestimated incident cases in late January and to a lesser extent they overestimated the number of cases in February and May. For all six surveys the median prediction for computational models and human judgment were both larger or smaller than the truth. Though the human judgement ensemble median prediction is at times closer to the truth than the compuational ensemble, the mean WIS score for individual predictions across all but one survey is smaller for computational models than for human judgement (Fig. 2B).

The median prediction of incident deaths was at times closer to the truth for computational models and at other times closer for a human judgement ensemble (Fig. 2C). January to May median predictions for computational models assumed a shallower decline in the number of deaths when compared to human judgement predictions for which the median prediction remained higher than the truth for predictions in January, February, and March, and then smaller than the truth in April. For one time point, the week beginning April 25th and ending May 1st, the median prediction from a computational ensemble was above the truth and the median predictions for human judgement was below the truth. Again, the mean WIS score for individual computational models is smaller when compared to human judgement, though the median prediction is at times closer to the truth for computational models and at times closer for human judgement (Fig. 2D)

### Pattern of missing forecasts for computational and human judgment models

**Fig. 3:**
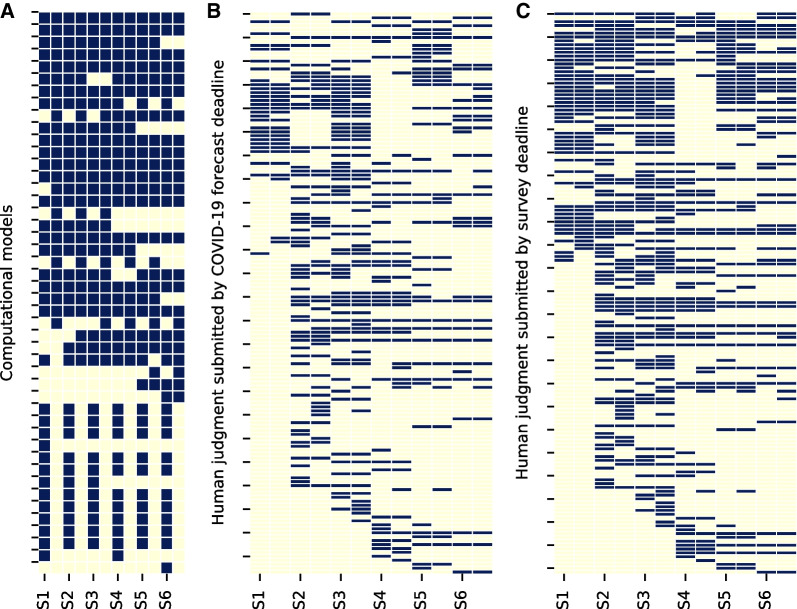
Submitted and missing forecasts made by **A** computational forecasts, ** B** human judgment forecasts submitted before the COVID-19 deadline, and **C** human judgment forecasts submitted by the survey deadline. Forecasts that were submitted are shown in blue and forecasts not submitted (missing) are shown in yellow. Rows represent a single model and columns are broken into six pairs—the left column (with the tick mark) corresponds to submissions of incident cases and the second column in the pair corresponds to submissions of incident deaths—which represent the six surveys from January 2021 to June 2021. The high proportion of missing forecasts made by human judgement models presents a methodological challenge when building a chimeric ensemble

The mean proportion of missing forecasts per model is higher for human judgment forecasts that submitted predictions at or before the forecast date set by the COVID-19 Forecast Hub (71%) versus computational models (34%): t-stat = 8.92, pvalue <0.001 (Fig. 3). The mean proportion of missing human judgment forecasts per model made by the survey deadline was smaller (66%) than was made by the COVID-19 Forecast Hub deadline (71%).

The proportion of surveys submitted by human judgment models compared to computational models that included both a prediction for cases and deaths was 23% vs 49%, that included a prediction for either cases or (exclusive) deaths is 11% vs 33%, and that did not submit both cases and deaths was 65% vs 17%.

### Comparison of a chimeric and computational ensemble and the impact of imputation

**Fig. 4:**
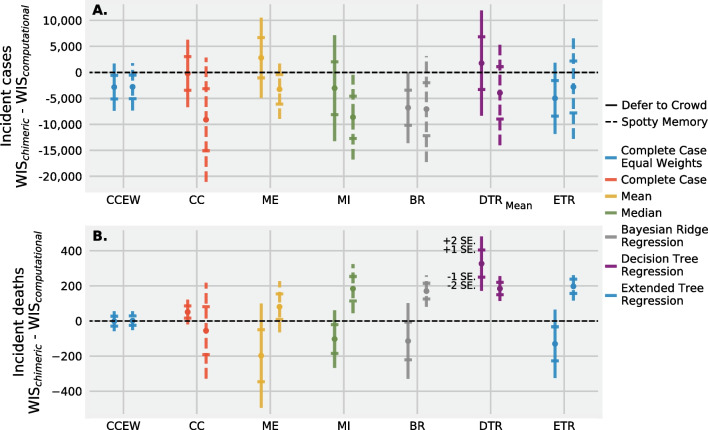
Mean difference in WIS for incident cases (**A**) and deaths (**B**) at the US national level between a chimeric ensemble and a computational ensemble paired across six different surveys from Jan 2021 to June 2021 for two strategies to impute missing values (“spotty memory” and “defer to the crowd”) and, within each strategy, 5 different techniques to impute missing forecasts. A chimeric ensemble—a combination of computational and human judgment models—improves WIS scores when the target is cases but weakens or maintains similar WIS scores when the target is deaths. There are negligible differences in mean WIS between a “defer to the crowd” and “spotty memory” imputation strategy for prediction of cases and a defer to the crowd approach appears to improve predictions compared to a spotty memory approach for predictions of incident deaths. Bayesian Ridge Regression (BR) and Median imputation (MI) are promising strategies to impute missing forecasts for incident cases

A chimeric ensemble improved predictions of incident cases compared to an computational model only ensemble. The mean WIS score assigned to predictions of incident cases for a chimeric ensemble minus the WIS score for a computational model paired by survey was negative (i.e. was improved) when using specific imputation techniques and strategies, and for the complete case (Fig. 4A). Imputing forecasts with a median imputation (MI) and “spotty memory” strategy had the smallest mean paired WIS score (mean: − 8624). Imputing missing predictions using a Bayesian ridge regression (BR) also performed well. A complete case equally weighted (CCEW in Fig. 4) chimeric ensemble reported similar predictive performance compared to an equally weighted computational ensemble using a “defer to the crowd” approach (mean, paired WIS: − 2835) and when using a “spotty memory” strategy (mean, paired WIS: -2,782). Weighting a combination of computational and human judgment models, coupled with an imputation strategy, may better predict incident cases at the US national level compared to a computational model only ensemble.

In contrast to incident cases, the paired mean WIS score for incident deaths was positive (i.e. performed worse) or close to zero for the majority of spotty memory imputation strategies, the complete case dataset, and a complete case data set where equal weights are assigned to all models, and were not significantly improved for the “defer to the crowd” strategy (Fig. 4B). A chimeric ensemble may not improve predictions of incident deaths compared to an ensemble of computational models alone.

### Performance based vs equal weighting

**Fig. 5:**
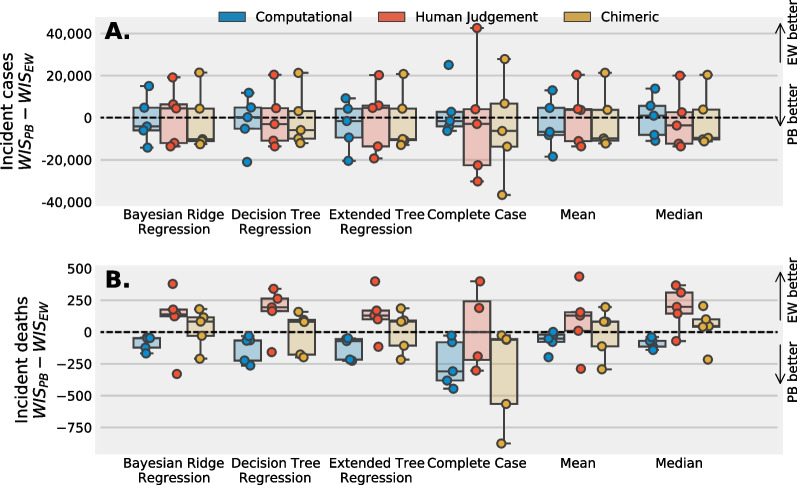
Median, 25th and 75th, and interquartile ranges for the difference between WIS scores when fitting a performance based ensemble (PB) and equally weighted ensemble (EW) paired by survey for three different ensembles: an ensemble that includes only computational models (blue), only human judgment (red), and a chimeric ensemble that includes both computational and human judgement models (gold). A “spotty memory” strategy was used along with five imputation techniques for training. Ensemble predictions are stratified by **A**  incident cases and **B** deaths. For the majority of imputation techniques used for predictions of incident cases, training a performance based ensemble shows similar results for a chimeric, computational, and human judgement ensemble. For deaths, performance based training improves predictions of a computational ensemble, shows little improvement to a chimeric ensemble, and weakens predictions of a human judgment ensemble

A performance based ensemble (PB) compared to assigning to all models equal weights (EW) decreases median WIS score for predictions of US national incident deaths when considering a computational ensemble, but not a chimeric or human judgement ensemble using a spotty memory imputation strategy. For all three ensembles WIS scores for predictions of cases show similar performance weights compared to equal weights  (Fig. 5).

**Fig. 6:**
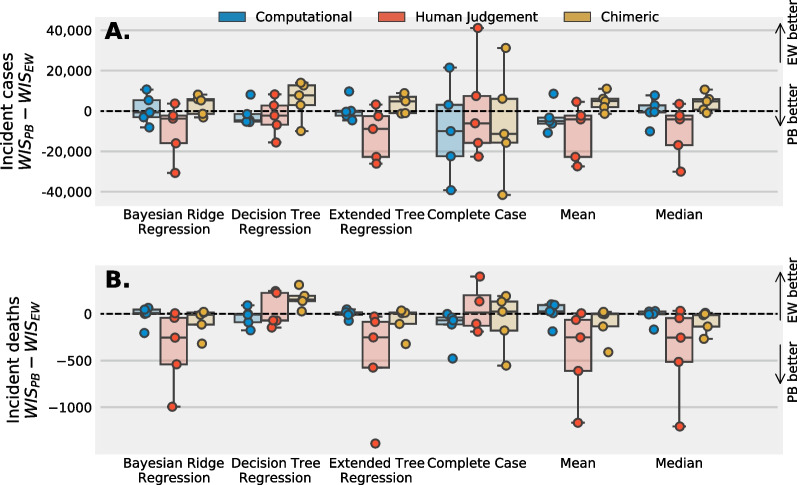
Median, 25th and 75th, and interquartile ranges for the difference between WIS scores when fitting a performance based ensemble (PB) and equally weighted ensemble (EW) paired by survey for three different ensembles: an ensemble that includes only computational models (blue), only human judgment (red), and a chimeric ensemble that includes both computational and human judgement models (gold). A “defer to the crowd” strategy was used along with five imputation techniques for training. Ensemble predictions are stratified by **A**  incident cases and **B** deaths. For the majority of imputation techniques used for predictions of incident cases, training a performance based ensemble improves the WIS score of a human judgement ensemble and weakens the performance of a computational and chimeric ensemble. For deaths, performance based training improves predictions of a a chimeric and human judgement ensemble, but for some imputation techniques weakens predictions of a computational ensemble. An algorithm that assigns different weights based on past performance, coupled with a “defer to the crowd” imputation strategy, may improve predictive performance of a chimeric ensemble

For predictions of incident cases with a spotty memory imputation strategy (Fig. 5A), the median difference in WIS score across all imputation techniques is negative, and the 25th to 75th percentiles include zero, indicating that performance based weighting is similar for predictions of incident cases. A defer to the crowd approach plus performance weighting improves predictions for a human judgement ensemble and for a computational ensemble, but weakens predictive performance for a chimeric ensemble (Fig. 6A).

For predictions of incident deaths, a performance based ensemble plus spotty memory approach improves WIS scores for a computational ensemble, shows similar performance for a chimeric ensemble, and weakens performance of a human judgement ensemble (Fig. 5B). A defer to the crowd approach plus performance weights improves human judgement and chimeric ensemble performance and weakens the performance of a computational ensemble (Fig. 6B) A complete case strategy plus performance weights shows similar WIS scores when using a human judgement and chimeric ensemble and improves predictions when using a computational and chimeric ensemble. The interquartile range for WIS_PB_–WIS_EW_ is above or covers zero for most chimeric and human judgment ensembles and is below zero for a computational ensemble when using a complete case approach.

## Chimeric ensemble’s ability to leverage human judgement

**Fig. 7:**
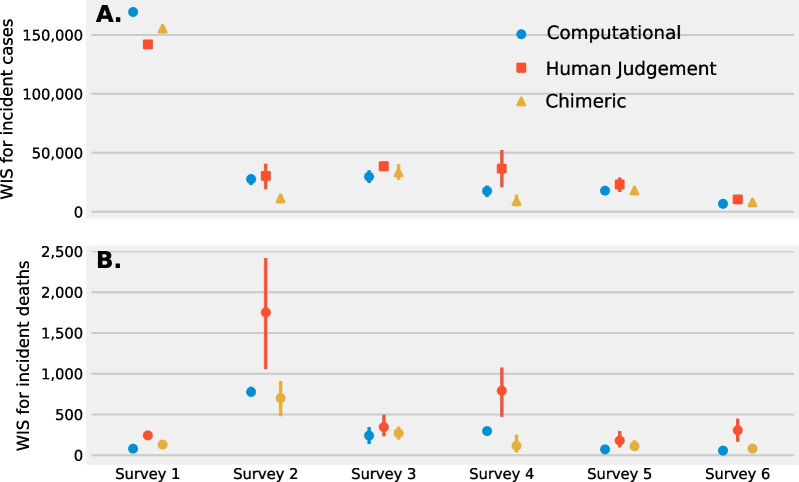
WIS scores for predictions of **A** incident cases and **B** incident deaths for a performance weighted computational ensemble (blue circle), human judgement ensemble (red square), and chimeric ensemble (yellow triangle) over all imputation techniques for a “defer to the crowd” imputation strategy. The mean WIS and 95% confidence interval over all imputation techniques is plotted. For incident cases, the predictive performance for a chimeric ensemble is similar to or improved when compared to a computational ensemble and despite poorer performance from human judgement alone. For incident deaths, though a computational ensemble has improved performance a chimeric ensemble outperforms a computational ensemble on two surveys and again is able to leverage human judgement to make improved forecasts

When stratified by survey, the WIS score for a chimeric ensemble’s prediction of incident cases is similar or improved vs a computational ensemble and, except for one survey, outperforms a human judgment ensemble (Fig. 7A). For incident deaths the WIS score is similar between a chimeric ensemble and computational ensemble. This similar predictive performance between the chimeric and computational ensemble is despite the poorer performing human judgement predictions in surveys two and four that are included in the chimeric ensemble (Fig. 7B).

## Discussion

We presented a first effort to combine direct probabilistic predictions of the spread and burden of an infectious agent generated by both computational models and human judgement.

A chimeric ensemble—a combination of forecasts generated by computational models and human judgment models—is capable of producing predictions that outperform an ensemble of computational models only. Though a chimeric ensemble has the potential to outperform a computational ensemble this is not always the case. Throughout these six surveys, a chimeric ensemble was also able to leverage at times poorer performing human judgement predictions to (i) outperform a computational ensemble and (ii) guard against relying too heavily on human judgement. Chimeric ensemble modeling is still in early stages and the reader should consider this work hypothesis generating.

There are several challenges to overcome when adding human judgment predictions.

Human judgment data must first be collected before predictions can be combined to produce a forecast. Data collection requires a team to pose questions to an audience of forecasters. Questions should be written as clear and concise as possible, to minimize bias, and written so that the forecaster understands how the truth will be determined (often called the resolution criteria). After questions are drafted they must be submitted to a prediction platform. A prediction platform should allow forecasters to easily view the question and resolution criteria, and allow the forecaster to submit their prediction with minimal effort. An immense amount of time and effort is needed to draft questions, and build and host a prediction platform. Organizing computational modeling efforts too requires an immense amount of effort to build [16, 95, 96]. However, the time needed to host computational efforts and answer questions throughout the prediction period may be less burdensome than with a human judgement platform.

After data collection there continue to be challenges with human judgment predictions. In our opinion, the most pressing issue is missing forecasts. Compared to computational models, we found that human forecasters have a much higher rate of missing forecast submissions, and if one wishes to use only models that submitted all forecasts (a complete case approach) it may not be feasible to include human judgment. Instead, an imputation strategy should be used to account for missing human judgment forecasts. Here we proposed two potential strategies to account for missing forecasts: a “defer to the crowd” and “spotty memory” approach, and we found that both methods resulted in similar predictive performance of incident cases and deaths for most imputation functions, though the “defer to the crowd” strategy may produce more accurate predictions of cases when using a bayesian regression function to impute missing values and a spotty memory approach produced the most accurate forecasts when using median imputation. Both methods were able to incorporate more human judgment models in an ensemble than a complete case analysis. That said, the chimeric ensemble using a complete case approach with equal weights—the most natural approach— showed improved performance compared to a computational ensemble and is one of the best pieces of evidence that adding human judgement can improve forecasts of an infectious agent.

An additional challenge when incorporating human judgement into an ensemble is the time needed to collect these human judgement forecasts (See Additional file 1: Fig. S5). We’ve found in this work that the majority of forecasts are collected close to when the survey closes. This is likely because forecasters wait to collect as much information about a question as possible until submitting a prediction. Though in this work the time to collect human judgment forecasts did not pose challenges to building an ensemble, this may pose a problem to future human judgement forecasting tasks that must produce forecasts rapidly.

The need to couple ensemble modeling with an imputation strategy is not unique to chimeric forecasts, but we feel the proportion of missing forecasts is unique [97]. Because the imputation strategies often fill in missing forecasts for a specific target with similar quantile values, one could consider the imputation approach we took to be a type of regularization and in past literature regularization was found to improve computational and human judgement ensembles [98, 99].

Whether to use a performance based or equal weighting for a chimeric ensemble is still unclear. A performance based chimeric ensemble compared to an equally weighted ensemble showed improved performance for some surveys and weakened performance for other surveys using a spotty memory approach (Additional file 1: Fig. S3), and showed improved performance as additional data was collected for a defer to the crowd approach coupled with a chimeric ensemble when predicting cases (Additional file 1: Fig. S4). A challenge when ensemble modeling, in addition to choosing an algorithm to assign different weights to models, is to know in advance whether or not differential weighting will improve predictive performance and whether or not human judgement will improve or weaken predictive performance. Some factors that may help determine if differential weighting is useful or if human judgement should be included could be the difference in predicted median between a computational ensemble and human judgement ensemble, or potentially the difference in uncertainty in predictions. More work should focus on a three step approach to ensemble modeling: (i) predicting whether human judgement will improve predictive performance, (ii) predicting if differential weighting would benefit a set of models, and (iii) then either choosing equal weights or differential weights.

A chimeric and human judgement ensemble’s ability to improve predictions of incident cases is consistent with past work studying predictions of exclusively human judgment [68]. Computational models often make more accurate predictions of deaths because they incorporate into their models reported cases, a signal for upcoming deaths. We are not sure whether or not humans considered the time series of incident cases when submitting predictions of deaths. Questions presented to forecasters did not suggest that cases could be a strong signal to consider when building a forecast for deaths. The question of how forecasters use time series information could lead to a controlled experiment to test human judgment’s ability to predict one time series by using a second, correlated time series. Previous literature suggests humans may make strong predictions that are short term, when there exists linear correlations between two concepts, and focus on information that most differed from their expectations [100–102]. But to the best of our knowledge no work has been done in the area of multi-cue probability theory and judgemental forecasting of time series by providing a second correlated time series.

Because the effort a human can spend on prediction is finite, and because of the above results that show human judgement improves predictions of cases the most, we recommend asking crowds to predict cases or similar targets that are strongly correlated to others (such as incident deaths) which may (i) improve predictions of cases and (ii) improve predictions of deaths if these human judgement predictions were used as input to a computational forecasting model.

This work has several limitations. We only evaluated twelve targets in common with the COVID-19 Forecast hub and so the results above should be considered exploratory rather than confirmatory. The limited number of targets brings up the broader limitation that human judgement cannot be applied to a large number of targets, locations, and forecast horizons like computational models. The ensemble model we chose to optimize average WIS was deterministic, made no attempt to regularize weights assigned to models, and is just one type of method to aggregate computational and human judgement models. The number of human judgement participants, while excellent, was still a limitation at times. The empirical nature of this work, versus a controlled laboratory experiment, as well makes it difficult to draw strong conclusions about the performance of human judgement, computational models, and their combined performance.

In the future we plan to focus on methodology: (i) by building more advanced ensemble algorithms to combine computational and human judgement models, (ii) methods to determine for which targets human judgement is needed and which targets it is not needed, (iii) imputation procedures that take into account the uncertainty when filling in missing forecasts, and (iv) strategies that allow the ensemble builder to preferentially assign higher weights to either humans or computational models perhaps via a prior distribution; data collection: (i) by proposing strategies to reduce the number of missing human judgement forecasts; explore the limits of human judgement: (i) by testing to what degree humans can use one time series to predict another, (ii) how humans construct mental models and generate predictions, and (iii) what additional information can human judgement provide that is supportive of public health efforts.

We envision a chimeric ensemble as a flexible aggregation technique that can manage and combine predictions throughout the evolution of an infectious agent and as a supportive tool for public health. A chimeric ensemble can begin to support primary and secondary preventive measures by relying on fast acting human judgment to forecast targets while data is collected and computational models are trained. Once computational models begin to forecast, a chimeric ensemble can integrate these forecasts with no down time. As computational models become accurate for specific targets then human judgement can be used to predict noisier targets which can be included in this type of ensemble.

## Supplementary Information

**Additional file 1.** A. Questions and Resolution Criteria. B. Forecasting platforms. C. List of included computational models form the COVID-19 Forecast Hub. D. Paired difference in WIS between a performance based and equally weighted ensemble across surveys. E. Counts of computational and human judgement models that submitted before the COVID-19 Forecast Hub deadlines.

## Data Availability

Human judgement consensus predictions and chimeric predictions of incident cases and incident death using an equally weighted ensemble approach are available for all surveys at the Zoltar Forecast Archive: https://zoltardata.com/model/511. Anonymized data on individual predictions is available upon request. Summary reports that were generated in real-time from January 2021 to June 2021 on all targets (not just cases and deaths) are available at https://github.com/computationalUncertaintyLab/aggStatModelsAndHumanJudgment_PUBL.
