# Supplementary material for: Chimeric forecasting: combining probabilistic predictions from computational models and human judgment
Source: BMC Infect Dis. 2022 Nov 10;22:833. doi: 10.1186/s12879-022-07794-5 (PMC9648897; doi:10.1186/s12879-022-07794-5)
Supplement: Supplementary file 1 — Additional file 1. A. Questions and Resolution Criteria. B. Forecasting platforms. C. List of included computational models form the COVID-19 Forecast Hub. D. Paired difference in WIS between a performance based and equally weighted ensemble across surveys. E. Counts of computational and human judgement models that submitted before the COVID-19 Forecast Hub deadlines. [file 12879_2022_7794_MOESM1_ESM.pdf]

Chimeric forecasting: combining probabilistic predictions from computational models and human judgment

Thomas McAndrew,[1,*] Allison Codi,[1] Juan Cambeiro,[2,3] Tamay Besiroglu,[2,4] David Braun,[5] Eva Chen,[6] Luis Enrique Urtubey De Cèsaris,[6] and Damon Luk[1]

[1]*College of Health, Lehigh University, Bethlehem, Pennsylvania, United States of America*

[2]*Metaculus, Santa Cruz, California, United States of America*

[3]*Department of Epidemiology, Mailman School of Public Health, Columbia University, New York, United States*

[4]*Massachusetts Institute of Technology, Cambridge, Massachusetts, United States*

[5]*Department of Psychology, Lehigh University, Bethlehem, Pennsylvania, United States of America*

[6]*Good Judgment Inc., New York, New York, United States of America*

(Dated: November 8, 2022)

* mcandrew@lehigh.edu

A. QUESTIONS AND RESOLUTION CRITERIA

A.1. Survey 01 (Open on 2021-01-06, Close on 2021-01-16)

- Question 1
  - Question: What will be the number of new incident U.S. adult and pediatric previous day admissions to the hospital with confirmed COVID-19 for the week beginning on 2021-01-24 and ending on 2021-01-30 (inclusive)?
  - Resolution Criteria:This question will resolve as the total number of adult plus pediatric previous day admissions with confirmed COVID-19 as recorded in the Department of Health and Human Service's report of COVID-19 reported patient impact and hospital capacity. The total previous day admissions is computed using two variables in this report: previous_day_admission_adult_covid_confirmed and previous_day_admission_pediatric_covid_confirmed. This report, and the resolution criteria, includes data on all 50 US states, Washington DC, Puerto Rico, and the US Virgin Islands (53 states and territories). The report will be accessed one week after the end of the month (2021-02-06).
  - Range:[0-240K]
  - Question URL: https://pandemic.metaculus.com/questions/6160/new-us-hospital-admissions-24-30-january/
- Question 2
  - Question: What will be the total number of new incident deaths due to COVID-19 in the US for the week beginning on 2021-01-24 and ending on 2021-01-30 (inclusive)?
  - Resolution Criteria: This question will resolve as the number of new deaths due to confirmed COVID-19 for the week beginning on 2021-01-24 and ending on 2021-01-30 (inclusive) as recorded in the Johns Hopkins University (JHU) CSSE Github data repository. This file records the daily number of deaths by county. From this file deaths are summed across all counties and aggregated by week to generate the number of new deaths per week. The report will be accessed one week after the end of the month (2021-02-06).
  - Range:[0-40k]
  - Question URL: https://pandemic.metaculus.com/questions/6161/new-us-covid-deaths-24-30-january/
- Question 3
  - Question: What will be the number of new incident confirmed positive COVID-19 cases in the US beginning on 2021-01-24 and ending on 2021-01-30 (inclusive)?
  - Resolution Criteria: This question will resolve as the number of new confirmed cases beginning on 2021-01-24 and ending on 2021-01-30 (inclusive) recorded in the Johns Hopkins University (JHU) CSSE Github data repository. This file records the daily number of cases by county. From this file cases are summed across all counties and aggregated by week to generate the number of new cases per week. The report will be accessed one week after the end of the month (2021-02-06).
  - Range:[0-3M]

  - Question URL: https://pandemic.metaculus.com/questions/6162/new-us-covid-cases-24-30-january/

- Question 4
  - Question: What factor should the median 4-week-ahead COVIDhub Ensemble forecast of national incident deaths made on 4 Jan(a forecast for the 24-30 Jan week) be multiplied by so that it equals the reported number of new US incident deaths?
  - Resolution Criteria: This question will resolve as the factor that the The COVID-19 Forecast Hub's "COVIDhub" Ensemble median forecast of the US national number of incident deaths 4 weeks into the future should be multiplied by (the reported number of incident deaths divided by the forecasted median) to equal the reported number of US national incident deaths as reported by the Johns Hopkins University (JHU) CSSE Github data repository.
  - Range:[0-3]
  - Question URL https://pandemic.metaculus.com/questions/6163/factor-covidhub-forecast-to-be-multiplied-by/

- Question 5
  - Question: What will be the cumulative number of deaths due to COVID-19 on 2021-12-31 if less than 50% of Americans initiate vaccination (1st dose received) with a COVID-19 vaccine by 2021-03-01?
  - Resolution Criteria: The percent of the population that received a COVID-19 vaccine on or before 2021-03-01 will be computed by dividing the number of individuals who have initiated vaccine (1st dose taken) provided by the CDC COVID data tracker by the current US population which on 2021-01-04 was reported to be 330,782,991 and multiplying this fraction by 100. The CDC COVID data tracker that counts the number of individuals who have initialized vaccination will be accessed when data is available after and as close as possible to 2021-03-01.

    To resolve deaths, we will use the cumulative number of deaths due to confirmed COVID-19 as recorded in the Johns Hopkins University (JHU) CSSE Github data repository. This file records the daily number of deaths by county. From this file deaths are summed across all counties and aggregated to week to generate the number of new deaths per week. The report will be accessed one week after 2021-12-31.

    9 January edit: This question will resolve ambiguously if greater than or equal to 50% of Americans are vaccinated by 2021-03-01.
  - Range:[0-1M]
  - Question URL: https://pandemic.metaculus.com/questions/6165/number-of-us-covid-deaths-if-50-vaccinated/

- Question 6
  - Question: What will be the cumulative number of deaths due to COVID-19 on 2021-12-31 if greater than or equal to 50% of Americans initiate vaccination (1st dose received) with a COVID-19 vaccine by 2021-03-01?
  - Resolution Criteria: The percent of the population that received a COVID-19 vaccine on or before 2021-03-01 will be computed by dividing the number of individuals who have initiated vaccine (1st dose taken) provided by the CDC COVID data tracker by the current US population which

on 2021-01-04 was reported to be 330,782,991 and multiplying this fraction by 100. The CDC COVID data tracker that counts the number of individuals who have initialized vaccination will be accessed when data is available after and as close as possible to 2021-03-01.

To resolve deaths, we will use the cumulative number of deaths due to confirmed COVID-19 as recorded in the Johns Hopkins University (JHU) CSSE Github data repository. This file records the daily number of deaths by county. From this file deaths are summed across all counties and aggregated to week to generate the number of new deaths per week. The report will be accessed one week after 2021-12-31.

9 January edit: This question will resolve ambiguously if less than 50% of Americans are vaccinated by 2021-03-01.

  - Range:[0-1M]
  - Question URL: https://pandemic.metaculus.com/questions/6164/number-of-covid-deaths-if-50-vaccinated/

- Question 7
  - Question: What will be the percent of B.1.1.7 among all S gene dropout SARS-CoV-2 samples submitted for genomic sequencing surveillance in the US between 2021-02-01 and 2021-02-15?
  - Resolution Criteria: This NextStrain page on S:N501Y as a proportion of overall S gene dropout samples in the US will be consulted for resolution. To access this data, scroll to the bottom of the page and download "selected metadata." In this Excel sheet, please consult the "Pangolin Lineage" column to find B.1.1.7 samples. Samples that have a "collection data" date between 2021-02-01 and 2021-02-15 (inclusive) will be considered. We will compute the proportion by dividing the number of B.1.1.7 samples by the total number of samples (which are all 69del samples) in the spreadsheet. NextStrain will be accessed in the last week of February (the week ending on 2021-02-27) for resolution.
  - Range:[0-100]
  - Question URL: https://pandemic.metaculus.com/questions/6166/-b117-among-all-s-gene-dropout-samples/

A.2. Survey 02 (Open on 2021-02-03, Close on 2021-02-15)

- Question 1
  - Question: What will be the number of new incident U.S. adult and pediatric admissions to the hospital with confirmed COVID-19 for the week beginning on 2021-02-21 and ending on 2021-02-27 (inclusive)?
  - Resolution Criteria: This question will resolve as the total number of adult plus pediatric previous day admissions with confirmed COVID-19 as recorded in the Department of Health and Human Service's report of COVID-19 reported patient impact and hospital capacity for the dates from 2021-02-22 to 2021-02-28, corresponding to the number of hospitalizations from 2021-02-21 to 2021-02-27. Daily updates are provided by the Department of Health and Human Services. The total previous day admissions is computed using two variables in this report: previous_day_admission_adult_covid_confirmed and previous_day_admission_pediatric_covid_confirmed and stored in Lehigh University's Computational Uncertainty Lab Github data repository. This

report, and the resolution criteria, includes data on all 50 US states, Washington DC, Puerto Rico, and the US Virgin Islands (53 states and territories). The report will be accessed no sooner than 2021-03-06.
  - Range:[0-200K]
  - Question URL Metaculus: https://pandemic.metaculus.com/questions/6468/new-us-covid-hospital-admissions-21-27-feb/
  - Question URL GJO: https://www.gjopen.com/questions/1921

- Question 2
  - What will be the total number of new incident deaths due to COVID-19 in the US for the week beginning on 2021-02-21 and ending on 2021-02-27 (inclusive)?
  - Resolution Criteria: This question will resolve as the number of new deaths due to confirmed COVID-19 for the week beginning on 2021-02-21 and ending on 2021-02-27 (inclusive) as recorded in the Johns Hopkins University (JHU) CSSE Github data repository. This file records the daily number of deaths by county. From this file deaths are summed across all counties and aggregated by week to generate the number of new deaths per week. The number of deaths for the week beginning on 2021-02-21 will be computed by adding the number of new deaths from the 2021-02-21 up to, and including, 2021-02-27. The report will be accessed no sooner than (2021-03-06).
  - Range:[0-30k]
  - Question URL Metaculus: https://pandemic.metaculus.com/questions/6466/new-us-covid-deaths-21-27-february/
  - Question URL GJO: https://www.gjopen.com/questions/1922

- Question 3
  - Question: What will be the total number of new incident confirmed positive COVID-19 cases in the US beginning on 2021-02-21 and ending on 2021-02-27 (inclusive)?
  - Resolution Criteria: This question will resolve as the number of new confirmed cases beginning on 2021-02-21 and ending on 2021-02-27 (inclusive) recorded in the Johns Hopkins University (JHU) CSSE Github data repository. This file records the daily number of cases by county. From this file cases are summed across all counties and aggregated by week to generate the number of new cases per week. The report will be accessed no sooner than 2021-03-06.
  - Range:[0-3M]
  - Question URL Metaculus: https://pandemic.metaculus.com/questions/6469/new-us-covid-cases-21-27-february/
  - Question URL GJO: https://www.gjopen.com/questions/1923

- Question 4
  - Question: What will be the cumulative number of people who have received one or more doses of a COVID-19 vaccine in the U.S. on 2021-02-28?
  - Resolution Criteria: This question will resolve as the cumulative number of people who receive one or more doses of a COVID-19 vaccine on 2021-02-28 as recorded by the Centers for Disease Control COVID-19 Data tracker in the column "Number of People Receiving 1 or More Doses." The dashboard is updated daily by 8pm ET and will be accessed on 2021-02-28 at approximately 10:00pm ET.

  - Range:[27M-85M]
  - Question URL Metaculus: https://pandemic.metaculus.com/questions/6472/cumulative-us-vaccinations-28-february/
  - Question URL GJO: https://www.gjopen.com/questions/1924

- Question 5
  - Question: How many variants of concern will be monitored by the US CDC as of 2021–03-07?
  - Resolution Criteria: This question will resolve as the number of variants of concern at the following link: "US COVID-19 Cases Caused by Variants" page as of Sunday, 2021–03-07. For example, as of 2021–02-02 this page shows that there are three variants: B.1.1.7, B.1.351, and P.1. This page is updated on Sundays, Tuesdays, and Thursdays by 7pm ET and will be accessed at approximately 10pm ET on 2021–03-07 (a Sunday).
  - Range:[0-8]
  - Question URL: https://pandemic.metaculus.com/questions/6474/-variants-monitored-by-cdc-on-7-march/

- Question 6
  - Question: What will be the percent of S:N501 sequences in the U.S. among all positive SARS-CoV-2 samples submitted to the GISAID database of genetic sequences between 2021-03-01 and 2021-03-07 (inclusive)?
  - Resolution Criteria: This question will resolve as the percentage of US S:N501 sequences among all positive SARS-CoV-2 samples submitted for genomic sequencing to the GISAID database between 2021-03-01 and 2021-03-07 (inclusive), as displayed on the "Distribution of S:N501 per country" plot on following website: https://covariants.org/variants/S.N501. This website pulls data from GISAID and makes it publicly accessible. This percentage will be accessed no sooner than 2021-03-15.
  - Range:[0-100]
  - Question URL: https://pandemic.metaculus.com/questions/6473/-sn501-in-us-for-week-of-1-march/

- Question 7
  - Question: What will be the percent of S:N501 sequences in the U.S. among all positive SARS-CoV-2 samples submitted to the GISAID database of genetic sequences between 2021-03-29 and 2021-04-04 (inclusive)?
  - Resolution Criteria: This question will resolve as the percentage of US S:N501 sequences among all positive SARS-CoV-2 samples submitted for genomic sequencing to the GISAID database between 2021-03-29 and 2021-04-04 (inclusive), as displayed on the "Distribution of S:N501 per country" plot on following website: https://covariants.org/variants/S.N501. This website pulls data from GISAID and makes it publicly accessible. This percentage will be accessed no sooner than 2021-04-12.
  - Range:[0-100]
  - Question URL: https://pandemic.metaculus.com/questions/6477/-sn501-in-us-for-week-of-29-march/

A.3. Survey 03 (Open on 2021-03-04, Close on 2021-03-15)

- Question 1
  - Question: What will be the number of new incident US adult and pediatric admissions to the hospital with confirmed COVID-19 for the week beginning on 2021-03-21 and ending on 2021-03-27 (inclusive)?
  - Resolution Criteria: This question will resolve as the total number of adult plus pediatric previous day admissions with confirmed COVID-19 as recorded in the Department of Health and Human Services report of COVID-19 reported patient impact and hospital capacity for the dates from 2021-03-22 to 2021-03-28, corresponding to the number of hospitalizations from 2021-03-21 to 2021-03-27. Daily updates are provided by the Department of Health and Human Services. The total previous day admissions is computed using two variables in this report: previous_day_admission_adult_covid_confirmed and previous_day_admission_pediatric_covid_confirmed and stored in Lehigh University's Computational Uncertainty Lab Github data repository. This report, and the resolution criteria, includes data on all 50 US states, Washington DC, Puerto Rico, and the US Virgin Islands (53 states and territories). The report will be accessed no sooner than (2021-04-04).
  - Range:[0-80K]
  - Metaculus question URL: https://pandemic.metaculus.com/questions/6712/new-us-covid-hospital-admissions-21-27-march/
  - GJO question URL: https://www.gjopen.com/questions/1952
- Question 2
  - What will be the total number of new incident deaths due to COVID-19 in the US for the week beginning on 2021-03-21 and ending on 2021-03-27 (inclusive)?
  - Resolution Criteria: This question will resolve as the number of new deaths due to confirmed COVID-19 for the week beginning on 2021-03-21 and ending on 2021-03-27 (inclusive) as recorded in the Johns Hopkins University (JHU) CSSE Github data repository. This file records the daily number of deaths by county. From this file deaths are summed across all counties and aggregated by week to generate the number of new deaths per week. The number of deaths for the week beginning on 2021-03-21 will be computed by adding the number of new deaths from the 2021-03-21 up to, and including, 2021-03-27. The report will be accessed no sooner than (2021-04-04).
  - Range:[0-25k]
  - Metaculus question URL: https://pandemic.metaculus.com/questions/6713/new-us-covid-deaths-21-27-march/
  - GJO question URL: https://www.gjopen.com/questions/1953
- Question 3
  - Question:What will be the number of new incident confirmed positive COVID-19 cases in the US beginning on 2021-03-21 and ending on 2021-03-27 (inclusive)?
  - Resolution Criteria: This question will resolve as the number of new confirmed cases beginning on 2021-03-21 and ending on 2021-03-27 (inclusive) recorded in the Johns Hopkins University (JHU) CSSE Github data repository. This file records the daily number of cases by county. From this

file cases are summed across all counties and aggregated by week to generate the number of new cases per week. The report will be accessed no sooner than 2021-04-04.
- Range:[0-2M]
- Metaculus question URL: https://pandemic.metaculus.com/questions/6714/new-us-covid-cases-21-27-march/
- GJO question URL: https://www.gjopen.com/questions/1954

- Question 4
  - Question: What will be the 7-day rolling average of the % B.1.1.7 in the US on 27 March 2021 (between 21 March 2021 and 27 March 2021)?
  - Resolution Criteria: This question will resolve as the 7 day rolling average of % sequences that are B.1.1.7 in the U.S. on 27 March 2021 (i.e. the average percentage between 21 March 2021 and 27 March 2021) at this website. This percentage will be accessed no sooner than 6 April 2021.
  - Range:[0-100]
  - Metaculus question URL https://pandemic.metaculus.com/questions/6717/-b117-in-the-us-21-27-march/

- Question 5
  - Question: In the context of community transmission, what will be the recommended minimum % of positive COVID-19 cases that should be sequenced?
  - Resolution Criteria: This question will resolve as the minimum CDC recommended percent of confirmed positive COVID-19 cases that should be sequenced that assumes community transmission. If the CDC does not release such guidance before the end of 2021, then the most-cited paper that provides a recommendation on the minimum recommended percent of positive COVID-19 cases that should be sequenced in the context of community transmission will be consulted on 1 January 2022.
  - Range:[0-100]
  - Metaculus question URL: https://pandemic.metaculus.com/questions/6718/-covid-cases-that-should-be-sequenced/

- Question 6
  - Question: How many variants of concern will be monitored by the US CDC as of 4 April?
  - Resolution Criteria: This question will resolve as the number of variants of concern monitored by the CDC as of Sunday, 2021-04-04. For example, as of 2021-03-02 this page shows that there are three variants: B.1.1.7, B.1.351, and P.1. This page is updated on Sundays, Tuesdays, and Thursdays by 7pm ET and will be accessed at approximately 10pm ET on 2021-04-04 (a Sunday).
  - Range:[0-8]
  - Metaculus question URL: https://pandemic.metaculus.com/questions/6719/-variants-monitored-by-cdc-on-4-april/

- Question 7
  - Question: What will be the cumulative number of people who receive one or more doses of a COVID-19 vaccine in the US on 2021-03-31?

  - Resolution Criteria: This question will resolve as the cumulative number of people who receive one or more doses of a COVID-19 vaccine on 2021-03-31 as recorded by the Centers for Disease Control COVID-19 Data tracker. The radio buttons ”People Receiving 1 or More Doses” and ”Cumulative” will be selected and the bar corresponding to 2021-03-31 will be accessed. Data is updated daily by 8pm ET and will be accessed no sooner than 2021-04-04. If the CDC changes how it reports vaccination data, we will provide clarifying language as necessary. For purposes of this question, a person receiving a single-dose vaccine would count as a person having received one or more doses of a COVID-19 vaccine.
  - Range:[0-140M]
  - Metaculus question URL: https://pandemic.metaculus.com/questions/6768/cumulative-1st-dose-us-vaccinations-31-march/
  - GJO question URL: https://www.gjopen.com/questions/1955

- Question 8
  - Question: What will be the cumulative number of people who receive two doses of a COVID-19 vaccine in the US on 2021-03-31?
  - Resolution Criteria: This question will resolve as the cumulative number of people who receive 2 doses of a COVID-19 vaccine on 2021-03-31 as recorded by the Centers for Disease Control COVID-19 Data tracker. The radio buttons ”People Receiving 2 Doses” and ”Cumulative” will be selected and the bar corresponding to 2021-03-31 will be accessed. Data is updated daily by 8pm ET and will be accessed no sooner than 2021-04-04. If the CDC changes how it reports vaccination data, we will provide clarifying language as necessary. For purposes of this question, a person receiving a single-dose vaccine would count as a person having received one or more doses of a COVID-19 vaccine.

    Mar 8 edit: On 2021-03-08, the CDC's vaccine tracker at https://covid.cdc.gov/covid-data-tracker/#vaccinations changed the ”receiving 2 doses” figure to ”fully vaccinated” to account for people who receive one dose of the Johnson & Johnson vaccine, which has been authorized as a single-dose regimen (by contrast, Pfizer/BioNTech and Moderna are authorized as two-dose vaccines). This question will resolve on the basis of the new ”fully vaccinated” figure reported by the CDC.
  - Range:[0-70M]
  - Metaculus question URL: https://pandemic.metaculus.com/questions/6769/cumulative-two-dose-us-vaccinations-31-march/

A.4. Survey 04 (Open on 2021-04-07, Close on 2021-04-20)

- Question 1
  - Question: What will be the number of new incident US adult and pediatric admissions to the hospital with confirmed COVID-19 for the week beginning on 2021-04-25 and ending on 2021-05-01 (inclusive)?

- Resolution Criteria: This question will resolve as the total number of adult plus pediatric previous day admissions with confirmed COVID-19 as recorded in the Department of Health and Human Services report of COVID-19 reported patient impact and hospital capacity for the dates from 2021-04-25 to 2021-05-01, corresponding to the number of hospitalizations from 2021-04-25 to 2021-05-01. Daily updates are provided by the Department of Health and Human Services. The total previous day admissions is computed using two variables in this report: previous_day_admission_adult_covid_confirmed and previous_day_admission_pediatric_covid_confirmed and stored in Lehigh University's Computational Uncertainty Lab Github data repository. This report, and the resolution criteria, includes data on all 50 US states, Washington DC, Puerto Rico, and the US Virgin Islands (53 states and territories). The report will be accessed no sooner than (2021-09-05).
- Range:[0-75K]
- Metaculus URL: https://pandemic.metaculus.com/questions/6985/new-us-covid-hospital-admissions-25-apr-1-may/
- GJO URL: https://www.gjopen.com/questions/1976

- Question 2
  - What will be the total number of new incident deaths due to COVID-19 in the US for the week beginning on 2021-04-25 and ending on 2021-05-01 (inclusive)?
  - Resolution Criteria: This question will resolve as the number of new deaths due to confirmed COVID-19 for the week beginning on 2021-04-25 and ending on 2021-05-01 (inclusive) as recorded in the Johns Hopkins University (JHU) CSSE Github data repository. This file records the daily number of deaths by county. From this file deaths are summed across all counties and aggregated by week to generate the number of new deaths per week. The number of deaths for the week beginning on 2021-04-25 will be computed by adding the number of new deaths from the 2021-04-25 up to, and including, 2021-05-01. The report will be accessed no sooner than 9 May 2021.
  - Range:[0-10k]
  - Metaculus URL: https://pandemic.metaculus.com/questions/6986/new-us-covid-deaths-25-apr-1-may/
  - GJO URL: https://www.gjopen.com/questions/1977

- Question 3
  - Question: What will be the number of new incident confirmed positive COVID-19 cases in the US beginning on 2021-04-25 and ending on 2021-05-01 (inclusive)?
  - Resolution Criteria: This question will resolve as the number of new confirmed cases beginning on 2021-04-25 and ending on 2021-05-01 (inclusive) recorded in the Johns Hopkins University (JHU) CSSE Github data repository. This file records the daily number of cases by county. From this file cases are summed across all counties and aggregated by week to generate the number of new cases per week. The report will be accessed no sooner than 2021-05-09.
  - Range:[0-1.2M]
  - Metaculus URL: https://pandemic.metaculus.com/questions/6987/new-us-covid-cases-25-apr-1-may/
  - GJO URL: https://www.gjopen.com/questions/1978

- Question 4
  - Question: What will be the cumulative number of people who receive one or more doses of a COVID-19 vaccine in the US on 2021-04-30?
  - Resolution Criteria: This question will resolve as the cumulative number of people who have received one or more doses of a vaccine on 2021-04-30 as recorded by the Centers for Disease Control COVID-19 Data tracker under Vaccine Trends. The dashboard is updated daily at 8pm ET and will be accessed no sooner than 9 May 2021.
  - Range:[109M-215M]
  - Metaculus URL `https://pandemic.metaculus.com/questions/6988/cumulative-1st-dose-us-vaccinations-30-april/`
  - GJO URL: `https://www.gjopen.com/questions/1979`
- Question 5
  - Question: What will be the cumulative number of people who are fully vaccinated against COVID-19 in the US on 2021-04-30?
  - Resolution Criteria: This question will resolve as the cumulative number of people who receive one or more doses of a COVID-19 vaccine on 2021-04-30 as recorded by the Centers for Disease Control COVID-19 Data tracker in the column "People Fully Vaccinated". The dashboard is updated daily at 8pm ET and will be accessed no sooner than 9 May 2021.
  - Range:[64M-130M]
  - Metaculus URL: `https://pandemic.metaculus.com/questions/6989/cumulative-fully-vaccinated-in-us-on-30-april/`
  - GJO URL: `https://www.gjopen.com/questions/1980`
- Question 6
  - Question: What will be the cumulative number of deaths in the US due to COVID-19 on 2021-12-31?
  - Resolution Criteria: This question will resolve as the number of cumulative deaths due to confirmed COVID-19 on 2021-12-31 as recorded in the Johns Hopkins University (JHU) CSSE Github data repository. This file records the daily number of deaths by county. The number of cumulative deaths at the end of the year will be computed by adding the cumulative number of deaths across states. This data, and the resolution criteria, includes data on all 50 US states, Washington DC, Puerto Rico, and the US Virgin Islands (53 states and territories). The report will be accessed no sooner than 9 January 2022.
  - Range:[555K-1.5M]
  - Metaculus URL: `https://pandemic.metaculus.com/questions/6990/cumulative-us-covid-deaths-by-end-of-2021/`
- Question 7
  - Question: What will be the 7-day rolling average of % B.1.1.7 in the US on 30 Apr 2021 (between 24 Apr 2021 and 30 Apr 2021)?

- Resolution Criteria: This question will resolve as the 7-day rolling average of % B.1.1.7 in the US (in other words, the frequency of B.1.1.7 as a percentage of all sequenced SARS-CoV-2 cases) on 30 Apr 2021 (the average percentage between 24 Apr 2021 and 30 Apr 2021) at the following website: https://outbreak.info/situation-reports?pango=B.1.1.7&loc=USA&selected=USA. This percentage will be accessed no sooner than 9 May 2021.
- Range:[0-100]
- Metaculus URL: https://pandemic.metaculus.com/questions/6991/-b117-in-us-on-30-april/

• Question 8

- Question:On what date will the United States CDC announce that they are tracking a SARS-CoV-2 variant that they classify as a variant of high consequence (VOHC)?
- Resolution Criteria: This question will resolve as the date that a SARS-CoV-2 variant is categorized under the ”Variant of High Consequence” section on the CDC's SARS-CoV-2 Variant Classifications and Definitions page.

  If no variant is classified as a VOHC before 1 January 2023, then this resolves as ¿ 31 December 2022.
- Range:[2021-04-07 - >2022-12-31]
- Metaculus URL: https://pandemic.metaculus.com/questions/6992/variant-of-high-consequence-before-2023

A.5. Survey 05 (Open on 2021-05-05, Close on 2021-05-18)

• Question 1

- Question: What will be the number of new incident U.S. adult and pediatric admissions to the hospital with confirmed COVID-19 for the week beginning on 2021-05-30 and ending on 2021-06-05 (inclusive)?
- Range:$[0 - 60K]$
- Metaculus URL: https://pandemic.metaculus.com/questions/7156/new-us-hospital-admissions-30-may-5-june/
- GJO URL: https://www.gjopen.com/questions/1996

• Question 2

- What will be the total number of new incident deaths due to COVID-19 in the US for the week beginning on 2021-05-30 and ending on 2021-06-05 (inclusive)?
- Resolution Criteria: This question will resolve as the number of new deaths due to confirmed COVID-19 for the week beginning on 2021-05-30 and ending on 2021-06-05 (inclusive) as recorded in the Johns Hopkins University (JHU) CSSE Github data repository. This file records the daily number of deaths by county. From this file deaths are summed across all counties and aggregated by week to generate the number of new deaths per week. The number of deaths for the week beginning on 2021-05-30 will be computed by adding the number of new deaths from the 2021-05-30 up to, and including, 2021-06-05. The report will be accessed no sooner than 2021-06-14.

  - Range:[$0 - 8K$]
  - Metaculus URL: https://pandemic.metaculus.com/questions/7157/new-us-covid-deaths-30-may-5-june/
  - GJO URL: https://www.gjopen.com/questions/1997

- Question 3
  - Question: What will be the number of new incident confirmed positive COVID-19 cases in the US beginning on 2021-05-30 and ending on 2021-06-05 (inclusive)?
  - Resolution Criteria: This question will resolve as the number of new confirmed cases beginning on 2021-05-30 and ending on 2021-06-05 (inclusive) recorded in the Johns Hopkins University (JHU) CSSE Github data repository. This file records the daily number of cases by county. From this file cases are summed across all counties and aggregated by week to generate the number of new cases per week. The report will be accessed on 2021-06-14.
  - Range:[$0 - 800K$]
  - Metaculus URL: https://pandemic.metaculus.com/questions/7158/new-us-covid-cases-30-may-5-june/
  - GJO URL: https://www.gjopen.com/questions/1998

- Question 4
  - Question: What will be the cumulative number of people who receive one or more doses of a COVID-19 vaccine in the US on 2021-05-31?
  - Resolution Criteria: This question will resolve as the cumulative number of people who have received one or more doses of a vaccine on 2021-05-31 as recorded by the Centers for Disease Control COVID-19 Data tracker under Vaccine Trends. The dashboard is updated daily at 8pm ET and will be accessed no sooner than 2021-06-14
  - Range:[$148M - 185M$]
  - Metaculus URL https://pandemic.metaculus.com/questions/7159/cumulative-1st-dose-us-vaccinations-31-may/
  - GJO URL: https://www.gjopen.com/questions/1999

- Question 5
  - Question: What will be the cumulative number of people who are fully vaccinated against COVID-19 in the US on 2021-05-31?
  - Resolution Criteria: This question will resolve as the cumulative number of people who receive one or more doses of a COVID-19 vaccine on 2021-05-31 as recorded by the Centers for Disease Control COVID-19 Data tracker in the column "People Fully Vaccinated". The dashboard is updated daily at 8pm ET and will be accessed no sooner than 2021-06-14.
  - Range:[$106M - 165M$]
  - Metaculus URL: https://pandemic.metaculus.com/questions/7160/cumulative-fully-vaccinated-in-us-on-31-may/
  - GJO URL: https://www.gjopen.com/questions/2000

- Question 6

- Question: What will be the cumulative number of deaths in the US due to COVID-19 on 2021-12-31?
- Resolution Criteria: This question will resolve as the number of cumulative deaths due to confirmed COVID-19 on 2021-12-31 as recorded in the Johns Hopkins University (JHU) CSSE Github data repository. This file records the daily number of deaths by county. The number of cumulative deaths at the end of the year will be computed by adding the cumulative number of deaths across states. This data, and the resolution criteria, includes data on all 50 US states, Washington DC, Puerto Rico, and the US Virgin Islands (53 states and territories). The report will be accessed no sooner than 2022-01-09.
- Range:$[575K - 1.1M]$
- Metaculus URL: https://pandemic.metaculus.com/questions/7161/cumulative-us-covid-deaths-by-end-of-2021/

• Question 7

- Question: What will be the % prevalence of SARS-CoV-2 variants thought to partially escape immunity for the two-week period 23 May - 05 Jun 2021?
- Resolution Criteria: This question will resolve on the basis of the first update that shows figures for the two-week period ending 05 Jun of the "Weighted Estimates of Proportions of SARS-CoV-2 Lineages" table on the U.S. CDC's "Variant Proportions" page. The percentages of variants that cause "reduced neutralization by convalescent and post-vaccination sera" will be added up. If between now and 05 Jun there are additional variants classified by the CDC as variants that cause "reduced neutralization by convalescent and post-vaccination sera," these will count toward the total percent figure. Likewise, if any of the variants that are currently classified as causing partial immune escape are removed from being classified as such, they will no longer count toward the total percent figure.
- Range:$[0 - 100]$
- Metaculus URL: https://pandemic.metaculus.com/questions/7164/prevalence-of-immune-evading-variants-5-june/

• Question 8

- Question:On what date will the United States CDC announce that they are tracking a SARS-CoV-2 variant that they classify as a variant of high consequence (VOHC)?
- Resolution Criteria: This question will resolve as the date that a SARS-CoV-2 variant is categorized under the "Variant of High Consequence" section on the CDC's SARS-CoV-2 Variant Classifications and Definitions page. If no variant is classified as a VOHC before 1 January 2025, then this resolves as $>$ 31 December 2024.
- Range:$[2021 - 05 - 01 - > 2024 - 12 - 31]$
- Metaculus URL: https://pandemic.metaculus.com/questions/7163/variant-of-high-consequence-before-2025/

A.6. Survey 6 (Open on 2021-06-02, Close on 2021-06-16)

• Question 1

  - Question: What will be the number of new incident U.S. adult and pediatric admissions to the hospital with confirmed COVID-19 for the week beginning on 2021-06-27 and ending on 2021-07-03 (inclusive)?
  - Resolution Criteria: This question will resolve as the total number of adult plus pediatric previous day admissions with confirmed COVID-19 as recorded in the Department of Health and Human Service report of COVID-19 reported patient impact and hospital capacity for the dates from 2021-06-28 to 2021-07-04, corresponding to the number of hospitalizations from 2021-06-27 to 2021-07-03. Daily updates are provided by the Department of Health and Human Services. The total previous day admissions is computed using two variables in this report: previous_day_admission_adult_covid_confirmed and previous_day_admission_pediatric_covid_confirmed and stored in Lehigh University's Computational Uncertainty Lab Github data repository. This report, and the resolution criteria, includes data on all 50 US states, Washington DC, Puerto Rico, and the US Virgin Islands (53 states and territories). The report will be accessed on 2021-07-12.
  - Range:[0-40K]
  - Metaculus URL: https://pandemic.metaculus.com/questions/7301/new-us-hospital-admissions-27-june-3-july/
  - GJO URL: https://www.gjopen.com/questions/2023

- Question 2
  - What will be the total number of new incident deaths due to COVID-19 in the US for the week beginning on 2021-06-27 and ending on 2021-07-03 (inclusive)?
  - Resolution Criteria: This question will resolve as the number of new deaths due to confirmed COVID-19 for the week beginning on 2021-06-27 and ending on 2021-07-03 (inclusive) as recorded in the Johns Hopkins University (JHU) CSSE Github data repository. This file records the daily number of deaths by county. From this file deaths are summed across all counties and aggregated by week to generate the number of new deaths per week. The number of deaths for the week beginning on 2021-06-27 will be computed by adding the number of new deaths from the 2021-06-27 up to, and including, 2021-07-03. The report will be accessed on 2021-07-12.
  - Range:[0-5k]
  - Metaculus URL: https://pandemic.metaculus.com/questions/7302/new-us-covid-deaths-27-june-3-july/
  - GJO URL: https://www.gjopen.com/questions/2024

- Question 3
  - Question: What will be the number of new incident confirmed positive COVID-19 cases in the US beginning on 2021-06-27 and ending on 2021-07-03 (inclusive)?
  - Resolution Criteria: This question will resolve as the number of new confirmed cases beginning on 2021-06-27 and ending on 2021-07-03 (inclusive) recorded in the Johns Hopkins University (JHU) CSSE Github data repository. This file records the daily number of cases by county. From this file cases are summed across all counties and aggregated by week to generate the number of new cases per week. The report will be accessed no sooner than 2021-07-12.
  - Range:[0-300K]
  - Metaculus URL: https://pandemic.metaculus.com/questions/7303/new-us-covid-cases-27-june-3-july/

  - GJO URL: https://www.gjopen.com/questions/2025

- Question 4
  - Question: What will be the cumulative number of people who receive one or more doses of a COVID-19 vaccine in the US on 2021-06-30?
  - Resolution Criteria: This question will resolve as the cumulative number of people who have received one or more doses of a vaccine on 2021-06-30 as recorded by the Centers for Disease Control COVID-19 Data tracker under Vaccine Trends. The dashboard is updated daily at 8pm ET and will be accessed no sooner than 2021-07-12.
  - Range:[169M-200M]
  - Metaculus URL https://pandemic.metaculus.com/questions/7305/cumulative-1st-dose-us-vaccinations-30-june/
  - GJO URL: https://www.gjopen.com/questions/2026

- Question 5
  - Question: What will be the cumulative number of people who are fully vaccinated against COVID-19 in the US on 2021-06-30?
  - Resolution Criteria: This question will resolve as the cumulative number of people who receive one or more doses of a COVID-19 vaccine on 2021-06-30 as recorded by the Centers for Disease Control COVID-19 Data tracker in the column People Fully Vaccinated. The dashboard is updated daily at 8pm ET and will be accessed no sooner than 2021-07-12.
  - Range:[136M-180M]
  - Metaculus URL: https://pandemic.metaculus.com/questions/7306/cumulative-fully-vaccinated-in-us-on-30-june/
  - GJO URL: https://www.gjopen.com/questions/2027

- Question 6
  - Question: What will be the cumulative number of deaths in the US due to COVID-19 on 2021-12-31?
  - Resolution Criteria: This question will resolve as the number of cumulative deaths due to confirmed COVID-19 on 2021-12-31 as recorded in the Johns Hopkins University (JHU) CSSE Github data repository. This file records the daily number of deaths by county. The number of cumulative deaths at the end of the year will be computed by adding the cumulative number of deaths across states. This data, and the resolution criteria, includes data on all 50 US states, Washington DC, Puerto Rico, and the US Virgin Islands (53 states and territories). The report will be accessed no sooner than 9 January 2022.
  - Range:[595K-1.2M]
  - Metaculus URL: https://pandemic.metaculus.com/questions/7307/cumulative-us-covid-deaths-by-end-of-2021/

- Question 7
  - Question: What will be the prevalence of SARS-CoV-2 variants thought to partially escape immunity for the two-week period 20 June - 03 July 2021?

- Resolution Criteria: This question will resolve on the basis of the first update that shows figures for the two-week period ending 03 July of the ”Weighted Estimates of Proportions of SARS-CoV-2 Lineages” table on the U.S. CDC’s ”Variant Proportions” page. The percentages of variants that cause ”reduced neutralization by convalescent and post-vaccination sera” will be added up. If between now and 03 July there are additional variants classified by the CDC as variants that cause ”reduced neutralization” by convalescent and/or post-vaccination sera, these will count toward the total percent figure. Likewise, if any of the variants that are currently classified as causing partial immune escape are removed from being classified as such, they will no longer count toward the total percent figure.
- Range:[0-100]
- Metaculus URL: https://pandemic.metaculus.com/questions/7308/prevalence-of-immune-evading-variants-3-july/

B. FORECASTING PLATFORMS

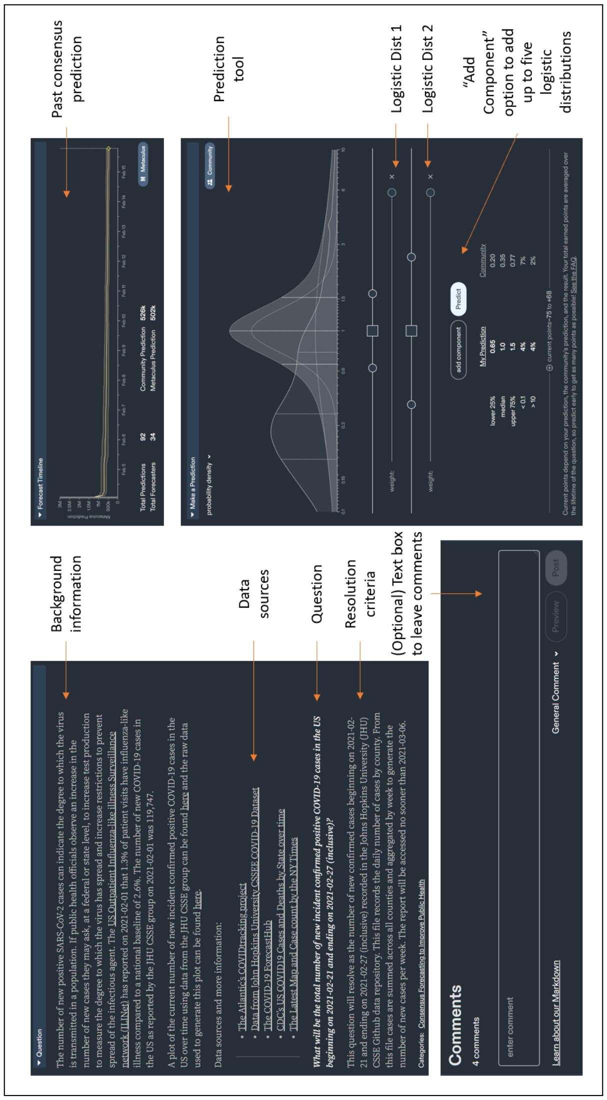

FIG. S1: The Metaculus format for submitting a forecast over a target of interest. Forecasters are presented with background information about the target and data sources that may be helpful when building a forecast. A forecaster can also view a consensus forecast from when the question was posed until present. A clearly defined question is asked in bold font and forecasters are also presented with the resolution criteria, how the ground truth for this question will be determined. Forecasters build a predictive distribution as a mixture of five logistic distributions. Forecasters, if they wish, can also share useful comments and questions with others in a chat box underneath their forecast.

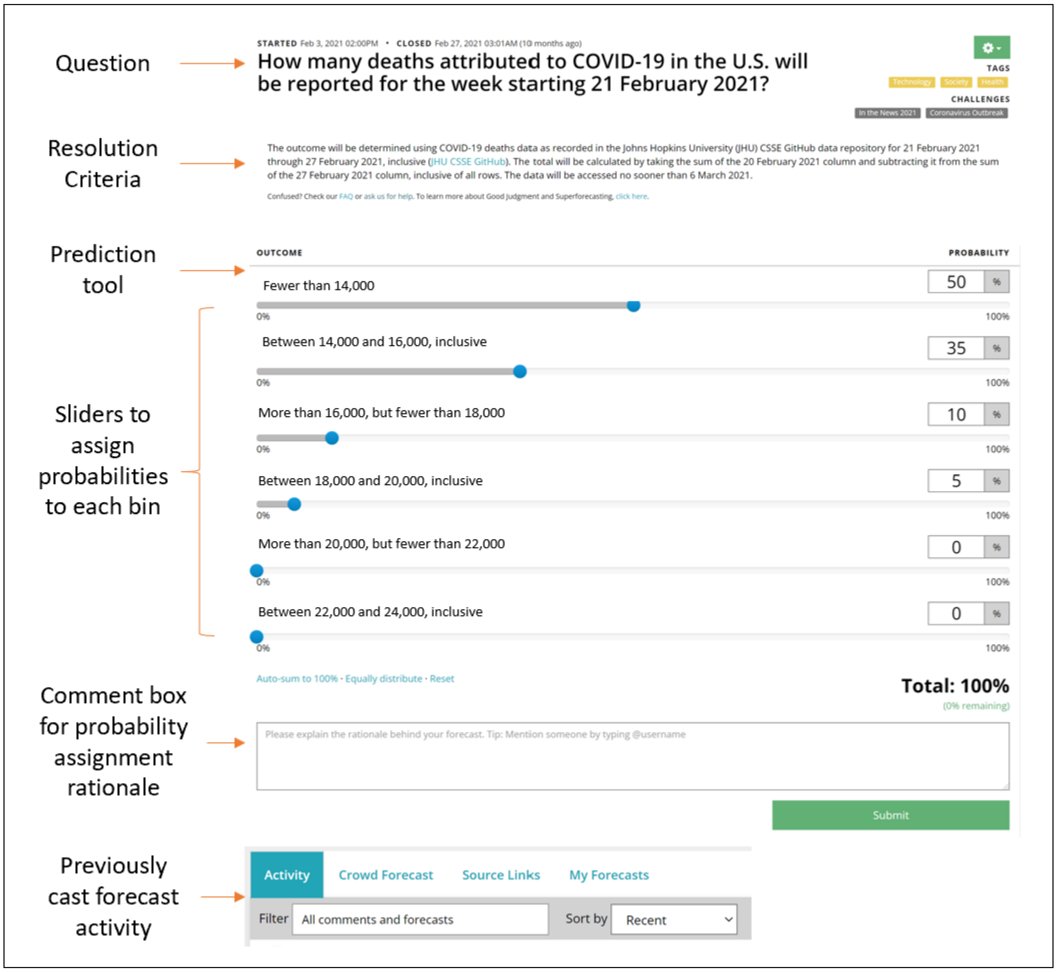

FIG. S2: The Good Judgment Open format for submitted a forecast over a target of interest. The question is posed to forecasters at the top of the page in bold font and underneath the question is the resolution criteria describing how the ground truth for this question will be determined. Forecasters can also view the current and past consensus distribution for this question. Forecasters build their predictive distribution by sliding N bars which represent intervals over possible truth values. Forecasters, if they wish, can also share useful comments and questions with others

C. LIST OF INCLUDED COMPUTATIONAL MODELS FORM THE COVID-19 FORECAST HUB

Model	Link to Data
BPagano-RtDriven	https://github.com/reichlab/covid19-forecast-hub/tree/master/data-processed/BPagano-RtDriven
CEID-Walk	https://github.com/reichlab/covid19-forecast-hub/tree/master/data-processed/CEID-Walk
Columbia_UNC-SurvCon	https://github.com/reichlab/covid19-forecast-hub/tree/master/data-processed/Columbia_UNC-SurvCon
Covid19Sim-Simulator	https://github.com/reichlab/covid19-forecast-hub/tree/master/data-processed/Covid19Sim-Simulator
CovidAnalytics-DELPHI	https://github.com/reichlab/covid19-forecast-hub/tree/master/data-processed/CovidAnalytics-DELPHI
COVIDhub-baseline	https://github.com/reichlab/covid19-forecast-hub/tree/master/data-processed/COVIDhub-baseline
CU-select	https://github.com/reichlab/covid19-forecast-hub/tree/master/data-processed/CU-select
DDS-NBDS	https://github.com/reichlab/covid19-forecast-hub/tree/master/data-processed/DDS-NBDS
IEM_MED-CovidProject	https://github.com/reichlab/covid19-forecast-hub/tree/master/data-processed/IEM_MED-CovidProject
IowaStateLW-STEM	https://github.com/reichlab/covid19-forecast-hub/tree/master/data-processed/IowaStateLW-STEM
JHU_IDD-CovidSP	https://github.com/reichlab/covid19-forecast-hub/tree/master/data-processed/JHU_IDD-CovidSP
JHUAPL-Bucky	https://github.com/reichlab/covid19-forecast-hub/tree/master/data-processed/JHUAPL-Bucky
Karlen-pypm	https://github.com/reichlab/covid19-forecast-hub/tree/master/data-processed/Karlen-pypm
LANL-GrowthRate	https://github.com/reichlab/covid19-forecast-hub/tree/master/data-processed/LANL-GrowthRate
LNQ-ens1	https://github.com/reichlab/covid19-forecast-hub/tree/master/data-processed/LNQ-ens1
Microsoft-DeepSTIA	https://github.com/reichlab/covid19-forecast-hub/tree/master/data-processed/Microsoft-DeepSTIA
OneQuietNight-ML	https://github.com/reichlab/covid19-forecast-hub/tree/master/data-processed/OneQuietNight-ML
QJHong-Encounter	https://github.com/reichlab/covid19-forecast-hub/tree/master/data-processed/QJHong-Encounter
RobertWalraven-ESG	https://github.com/reichlab/covid19-forecast-hub/tree/master/data-processed/RobertWalraven-ESG
SigSci-TS	https://github.com/reichlab/covid19-forecast-hub/tree/master/data-processed/SigSci-TS
UCF-AEM	https://github.com/reichlab/covid19-forecast-hub/tree/master/data-processed/UCF-AEM
UCLA-SuEIR	https://github.com/reichlab/covid19-forecast-hub/tree/master/data-processed/UCLA-SuEIR
UMich-RidgeTfReg	https://github.com/reichlab/covid19-forecast-hub/tree/master/data-processed/UMich-RidgeTfReg

UpstateSU-GRU	https://github.com/reichlab/covid19-forecast-hub/tree/master/data-processed/UpstateSU-GRU
USC-SI_kJalpha	https://github.com/reichlab/covid19-forecast-hub/tree/master/data-processed/USC-SI_kJalpha
UVA-Ensemble	https://github.com/reichlab/covid19-forecast-hub/tree/master/data-processed/UVA-Ensemble
JHU_CSSE-DECOM	https://github.com/reichlab/covid19-forecast-hub/tree/master/data-processed/JHU_CSSE-DECOM
MIT_ISOLAT-Mixtures	https://github.com/reichlab/covid19-forecast-hub/tree/master/data-processed/MIT_ISOLAT-Mixtures
MOBS-GLEAM_COVID	https://github.com/reichlab/covid19-forecast-hub/tree/master/data-processed/MOBS-GLEAM_COVID
FRBSF_Wilson-Econometric	https://github.com/reichlab/covid19-forecast-hub/tree/master/data-processed/FRBSF_Wilson-Econometric
MUNI-ARIMA	https://github.com/reichlab/covid19-forecast-hub/tree/master/data-processed/MUNI-ARIMA
MIT-Cassandra	https://github.com/reichlab/covid19-forecast-hub/tree/master/data-processed/MIT-Cassandra
epiforecasts-ensemble1	https://github.com/reichlab/covid19-forecast-hub/tree/master/data-processed/epiforecasts-ensemble1
GT-DeepCOVID	https://github.com/reichlab/covid19-forecast-hub/tree/master/data-processed/GT-DeepCOVID
MIT_CritData-GBCF	https://github.com/reichlab/covid19-forecast-hub/tree/master/data-processed/MIT_CritData-GBCF
MITCovAlliance-SIR	https://github.com/reichlab/covid19-forecast-hub/tree/master/data-processed/MITCovAlliance-SIR
OliverWyman-Navigator	https://github.com/reichlab/covid19-forecast-hub/tree/master/data-processed/OliverWyman-Navigator
PSI-DRAFT	https://github.com/reichlab/covid19-forecast-hub/tree/master/data-processed/PSI-DRAFT
RPI_UW-Mob_Collision	https://github.com/reichlab/covid19-forecast-hub/tree/master/data-processed/RPI_UW-Mob_Collision
SteveMcConnell-CovidComplete	https://github.com/reichlab/covid19-forecast-hub/tree/master/data-processed/SteveMcConnell-CovidComplete
UA-EpiCovDA	https://github.com/reichlab/covid19-forecast-hub/tree/master/data-processed/UA-EpiCovDA
UCM_MESALab-FoGSEIR	https://github.com/reichlab/covid19-forecast-hub/tree/master/data-processed/UCM_MESALab-FoGSEIR
UCSD_NEU-DeepGLEAM	https://github.com/reichlab/covid19-forecast-hub/tree/master/data-processed/UCSD_NEU-DeepGLEAM
UMass-MechBayes	https://github.com/reichlab/covid19-forecast-hub/tree/master/data-processed/UMass-MechBayes
UT-Mobility	https://github.com/reichlab/covid19-forecast-hub/tree/master/data-processed/UT-Mobility
IHME-CurveFit	https://github.com/reichlab/covid19-forecast-hub/tree/master/data-processed/IHME-CurveFit

D. PAIRED DIFFERENCE IN WIS BETWEEN A PERFORMANCE BASED AND EQUALLY WEIGHTED ENSEMBLE ACROSS SURVEYS

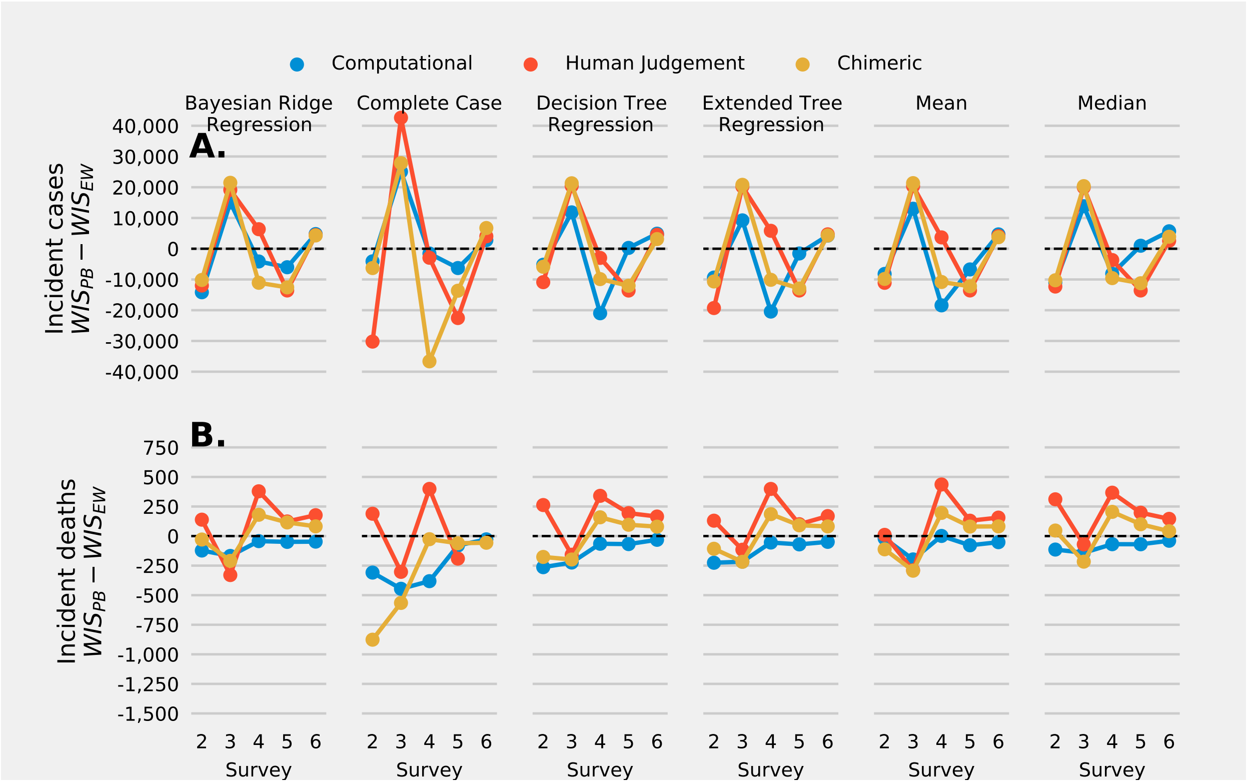

FIG. S3: Paired difference in WIS score for predictions of incident cases (A.) and deaths (B.) for surveys 2, 3, 4, 5, 6 between a performance based and equally weighted computational ensemble (blue), human judgement (red), and chimeric ensemble (gold) using a "spotty memory" imputation approach.

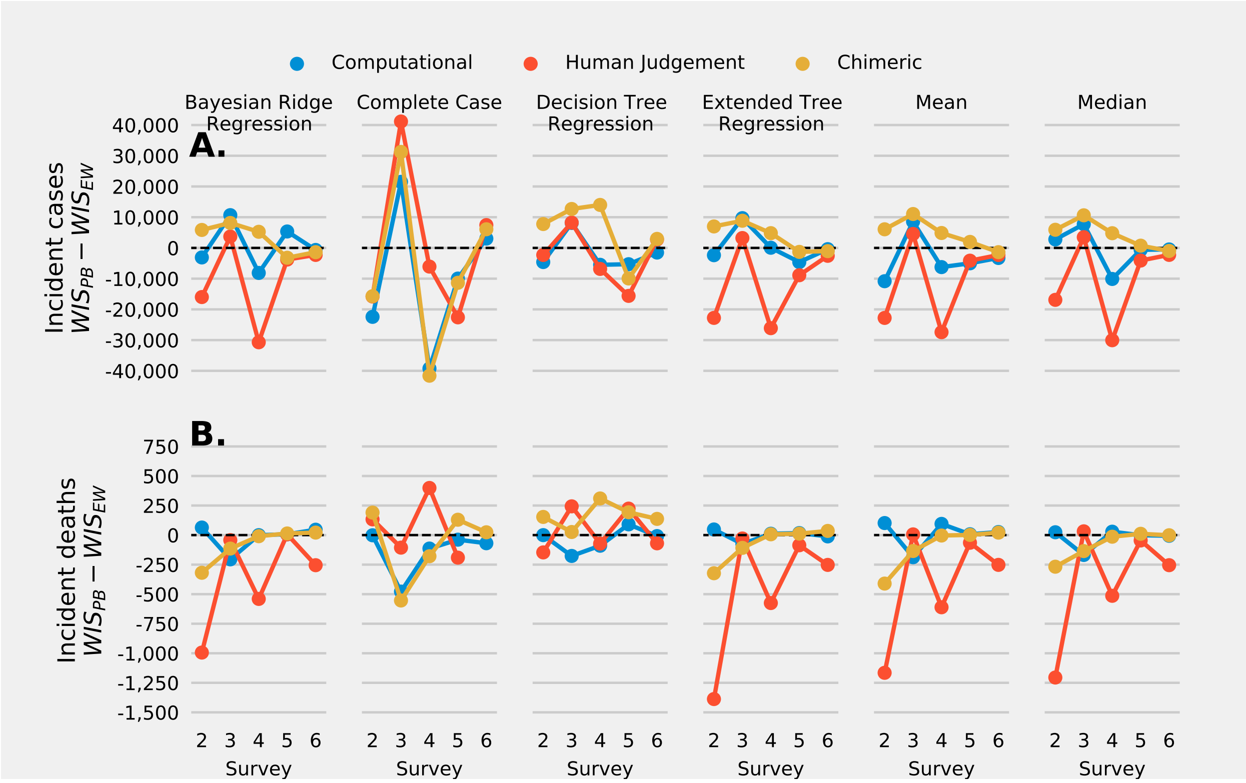

FIG. S4: Paired difference in WIS score for predictions of incident cases (A.) and deaths (B.) for surveys 2, 3, 4, 5, 6 between a performance based and equally weighted computational ensemble (blue), human judgement (red), and chimeric ensemble (gold) using a ”defer to the crowd” imputation approach.

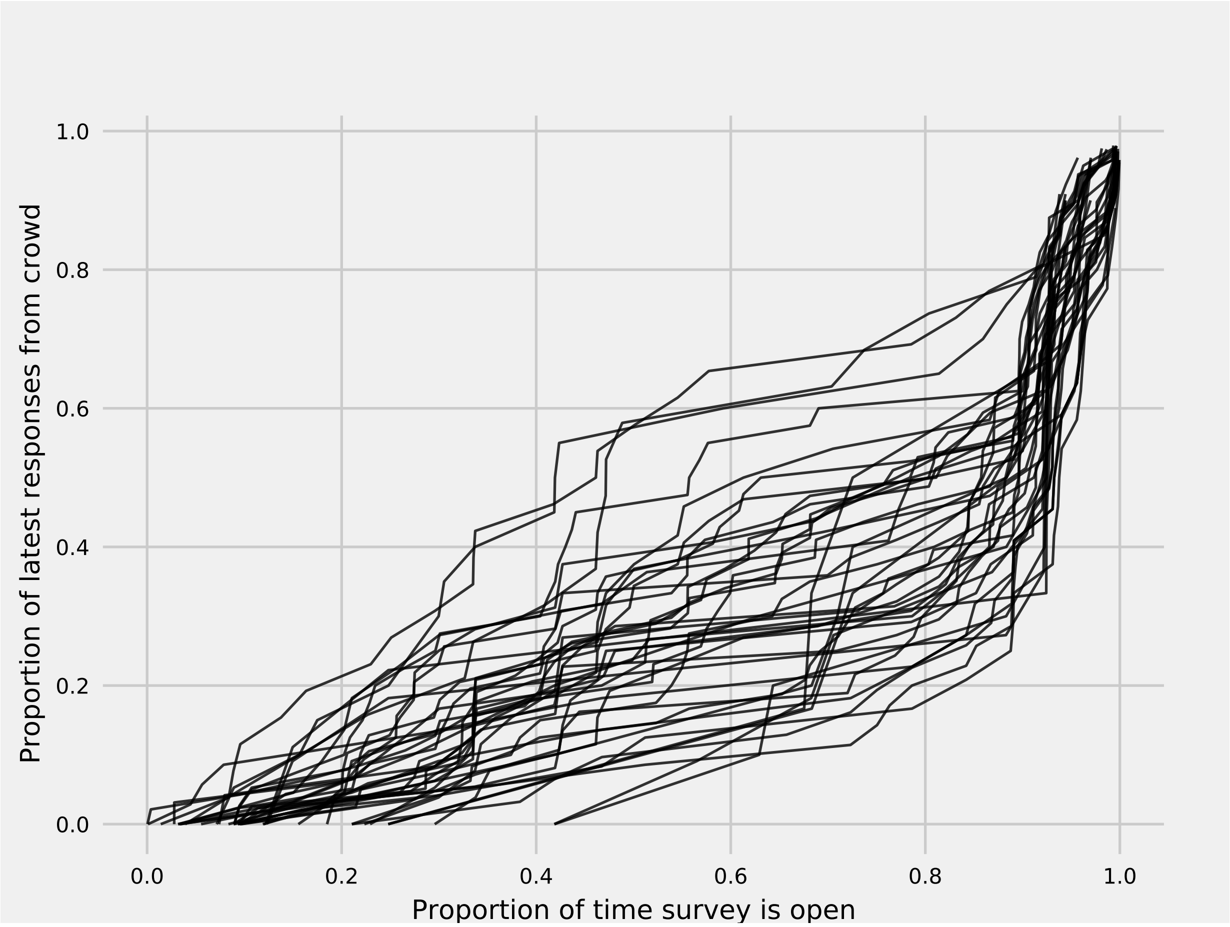

FIG. S5: The cumulative proportion of individuals who submitted a forecast to a question posed across all six surveys. The horizontal axis is the time an individual submitted a forecast. A time of zero represents the time the question was open for submission and a time of one represents the time that the survey was closed.

E. COUNTS OF COMPUTATIONAL AND HUMAN JUDGEMENT MODELS THAT SUBMITTED BEFORE THE COVID-19 FORECAST HUB DEADLINES

Survey	Target	Computational models	Metaculus	GJO
1	Cases	25	22	00
2	Cases	27	14	35
3	Cases	27	20	40
4	Cases	24	06	32
5	Cases	24	15	21
6	Cases	23	14	19
1	Deaths	34	24	00
2	Deaths	34	13	42
3	Deaths	34	22	39
4	Deaths	34	05	34
5	Deaths	33	16	24
6	Deaths	33	13	17

TABLE II: Counts of computational and human judgement models that submitted predictions of cases and deaths by the COVID-19 Forecast hub deadline stratified by survey
